# *PMEPA1* Is a Prognostic Biomarker That Correlates With Cell Malignancy and the Tumor Microenvironment in Bladder Cancer

**DOI:** 10.3389/fimmu.2021.705086

**Published:** 2021-10-28

**Authors:** Dongxu Qiu, Jian Hu, Jiao Hu, Anze Yu, Belaydi Othmane, Tongchen He, Jian Ding, Xu Cheng, Wenbiao Ren, Xiyan Tan, Qiaoyan Yu, Jinbo Chen, Xiongbing Zu

**Affiliations:** ^1^ Department of Urology, Xiangya Hospital, Central South University, Changsha, China; ^2^ Department of Urology, Central People’s Hospital of Zhanjiang, Zhanjiang, China; ^3^ Immunobiology & Transplant Science Center, Houston Methodist Research Institute, Texas Medical Center, Houston, TX, United States; ^4^ Institute for Infection Prevention and Hospital Epidemiology, Medical Center, University of Freiburg, Freiburg, Germany; ^5^ George Whipple Lab for Cancer Research, Departments of Pathology and Urology, and The Wilmot Cancer Institute, University of Rochester Medical Center, Rochester, NY, United States; ^6^ Center for Molecular Medicine, Xiangya Hospital, Central South University, Changsha, China

**Keywords:** bladder cancer, prostate transmembrane protein androgen induced 1, tumor microenvironment, prognostic biomarker, immune infiltration

## Abstract

Prostate transmembrane protein androgen induced 1 (*PMEPA1*) has been reported to promote cancer progression, but the potential role of *PMEPA1* in bladder cancer (BLCA) remains elusive. We assess the role of *PMEPA1* in BLCA, *via* a publicly available database and *in vitro* study. *PMEPA1* was identified from 107 differentially expressed genes (DEGs) to have prognostic value. GO, KEGG, and GSEA analysis indicated that *PMEPA1* was involved in cancer progression and the tumor microenvironment (TME). Then bioinformatical analysis in TCGA, GEO, TIMER, and TISIDB show a positive correlation with the inflammation and infiltration levels of three tumor-infiltrating immune cells (TAMs, CAFs, and MDSCs) and immune/stromal scores in TME. Moreover, *in vitro* study revealed that *PMEPA1* promotes bladder cancer cell malignancy. Immunohistochemistry and survival analysis shed light on *PMEPA1* potential to be a novel biomarker in predicting tumor progression and prognosis. At last, we also analyzed the role of *PMEPA1* in predicting the molecular subtype and the response to several treatment options in BLCA. We found that *PMEPA1* may be a novel potential biomarker to predict the progression, prognosis, and molecular subtype of BLCA.

## Introduction

BLCA is one of the main causes of mortality and morbidity all over the world, with about 380,000 new cases and 150,000 deaths per year (1). All initial diagnosed cases can be divided into non-muscle-invasive bladder cancer (NMIBC) and muscle-invasive bladder cancer (MIBC) disease. Bladder cancer with invasion of the smooth muscle layer around the bladder is diagnosed as MIBC (T2, T3, and T4) and consists of about 20% of all cases. MIBC is characterized by a high risk (about 40% - 60%) of metastasis and a poor prognosis (2). Moreover, if not detected and treated properly, at least one-third of NMIBC which are relatively easy to deal with ultimately invade the bladder wall and metastasize into neighboring organs or lymph nodes by undergoing radical molecular and cellular changes (2, 3). This diversity in somatic mutations exhibited by MIBC leads to great variability in cancer aggressiveness, progression, and response rates making MIBC particularly difficult to treat (4). Therefore, finding DEGs between NMIBC and MIBC tumors and identifying the potential biomarkers may help us reduce the invasion rate of NMIBC and even find novel therapeutics to adequately fight against MIBC.

Prostate transmembrane protein androgen induced 1 (*PMEPA1*) is a multifunctional protein and has very important values in prostate tumorigenesis. *PMEPA1* has a growth-promoting or inhibitory role in prostate cancer, depending on whether the cancer cells are negative or positive to androgen receptor (AR) (5, 6). It can be up-regulated by transforming growth factor (TGF) while inhibiting TGF signaling through a negative feedback loop. In addition, the *PMEPA1* gene has been shown to induce tumorigenesis *via* interfering with other signaling cascades such as mutated p53, Hippo signaling, Wnt, and EGF (7–14). Moreover, *PMEPA1* is a potential tumor suppressor in multiple myeloma (MM) and induces MM cell apoptosis by mediating c-Maf polyubiquitination and degradation but independently of the TGF-β signaling (15). However, the function of *PMEPA1* in BLCA has not been identified.

Increasing evidence suggests that the tumor microenvironment (TME) plays an important role in tumor progression and metastasis (16, 17). The tumor microenvironment is composed of cancer cells and various types of nonmalignant cells such as myeloid-derived suppressor cells (MDSC), tumor-associated macrophages (TAMs), cancer-associated fibroblasts (CAFs) and immune cells. Among those nontumor cells, stromal cells and Immune cells take a critical place in the whole process of tumors from genesis to transferring, and have a certain value in clinical diagnosis and prognosis. In previous studies, an algorithm named ESTIMATE designed by Yoshihara et al, was applied to determine the expression level of certain genes of stromal and immune cells by calculating stromal and immune scores to estimate these cells’ proportion and predict the infiltration of nonmalignant cells (18, 19)

## Materials and Methods

### Data Retrieval and Preprocessing

The Cancer Genome Atlas (TCGA) data: The mRNA sequencing expression profile and survival information of patients with BLCA were downloaded from the UCSC Xena data portal (20). Gene Expression Omnibus (GEO): five BLCA GEO cohorts were downloaded, namely GSE13507, GSE31684, GSE48075, GSE32894, and GSE32548. Microarray data of GSE32894, GSE48075, and GSE32548 were all on account of GPL6947 Platforms (Illumina HumanHT-12 V3.0 expression beadchip), which included 93 MIBC tissues and 213 NMIBC tissues, 72 MIBC tissues and 67 NMIBC tissues and 38 MIBC tissues and 92 NMIBC tissues, respectively.

### Patients

All human experiments were approved by the Medical Ethics Committee of Xiangya Hospital of Central South University, and informed consent was obtained from all subjects. There were 60 bladder cancer samples, among which 11 were female and 49 were male; 21 were 30-60 years old and 39 were 60-90 years old (**Table S1**). Pathologic diagnoses were evaluated by pathologists *via* biopsy reports.

### Identification of *PMEPA1 via* Bioinformatical Analyses of the GEO and TCGA Databases

DEGs between MIBC and NMIBC in 3 GEO datasets (GSE32894, GSE48075, and GSE32548) were identified *via* GEO2R online tools (https://www.ncbi.nlm.nih.gov/geo/query/acc.cgi?acc=GSE48075) (21) with |logFC| > 1 and adjust P value < 0.05. Then, the DEGs that 3 GEO datasets have in common were acquired *via* Venn software online (http://bioinformatics.psb.ugent.be/webtools/Venn/). The DEGs with log FC < 0 and log FC > 0 were separately considered down-regulated genes and up-regulated gene. Volcano plots were plotted by ggplot2 R package. Gene Ontology (GO) and Kyoto Encyclopedia of Genes and Genomes (KEGG) pathway analyses were applied *via* Cytoscape software 3.6.1 and ClueGO v2.5.7. For Kaplan–Meier curves, p-values and hazard ratio (HR) with 95% confidence interval (CI) were generated by log-rank tests and univariate Cox proportional hazards regression. All data of normal tissue samples were obtained from BLCA in the GTEx V8 release version (https://gtexportal.org/home/datasets). Complete description of the donor genders, multiple ethnicity groups, wide age range, the biospecimen procurement methods and sample fixation were described in GTEx official annotation (22, 23).

### Significant Pathways Influenced by *PMEPA1* in TCGA and GSE31684

DEGs between high- and low-*PMEPA1* group in TCGA were identified *via* Limma package (version: 3.40.2) of R software (20) with |logFC| > 1 and adjust P value < 0.05. GO and KEGG analyses were performed to analyze the top 200 down-regulated DEGs. Furthermore, gene set enrichment analysis (GSEA) was performed to analyze the GSE31684 dataset (including 279 patients with bladder cancer divided into high- and low-*PMEPA1* groups) *via* GSEA soft-ware 3.0 from the Broad Institute (24). Nominal P-value < 0.05 and false discovery rate (FDR) < 0.2 were considered statistically significant.

### TISIDB and TIMER Analysis

TISIDB (http://cis.hku.hk/TISIDB/) is a web portal for tumor and immune system interaction, which integrates multiple heterogeneous data types. Correlation between *PMEPA1* and 124 immunomodulators (chemokines, MHC-s, immune stimulators and receptors) and 28 tumor-associated immune cells were determined using the TISIDB (25). SIGLEC15, IDO1, CD274, HAVCR2, PDCD1, CTLA4, LAG3 and PDCD1LG2 are transcripts related to immune checkpoints (26).

The relationship between *PMEPA1* expression and tumor-infiltrating immune cells (TIICs) in 32 cancer types was determined using the TIMER (https://cistrome.shinyapps.io/timer/) (27). TIMER infers the abundance of TIICs applying the statistical analysis of gene expression profiles (28). We analyzed the association between the level of *PMEPA1* gene expression and the abundance of infiltrating immune cells, including tumor-associated macrophages (TAMs), cancer-associated fibroblast (CAFs), myeloid-derived suppressor cells (MDSCs), monocytes, neutrophils, Tregs, myeloid dendritic cells (DCs), NK cells, B cells, CD4+ T cells, and CD8+ T cells based on the expression of specific marker genes in different cancers including BLCA.

The marker genes used for the analysis of the tumor-infiltrating immune cells including T cells, B cells, monocytes, TAMs, M1 macrophages, M2 macrophages, neutrophils, natural killer (NK) cells, dendritic cells (DCs), T-helper (Th) cells, follicular helper T (Tfh) cells, T-helper 17 (Th17) cells, Tregs, cancer-associated fibroblasts (CAFs), and myeloid-derived suppressor cells (MDSCs) were based on data from previous studies (29–36).

### The Effect of *PMEPA1* on TME in Three Datasets (GSE32894, GSE48075, and GSE13507)

The immune scores, stromal scores, and ESTIMATE scores were calculated using the ESTIMATE R package. The infiltration scores of TAMs and cancer-associated fibroblasts (CAFs) were calculated using CIBERSORT and MCPcounter R package. We identified the effector genes of inflammatory cytokines from previous studies (37). Heatmaps were plotted by http://www.bioinformatics.com.cn, an online platform for data analysis and visualization. The correlation between *PMEPA1* and 38 immune genes (immune checkpoints, marker genes of TAMs, CAFs, MDSCs, and inflammatory cytokines) was calculated using the psych R package. The scatterplots were plotted by GraphPad Prism7.

### Survival Analyses of Clinical Parameters in Three Datasets

Kaplan–Meier curves, univariate and subgroup analyses of overall survival (OS) were performed using the GraphPad Prism7. Multivariable Cox regression models were built using SPSS. Specifically, univariate analyses were performed for the available clinical parameters, along with the respective *PMEPA1* expression data and calculated immune scores, and only significant factors (with p < 0.05) from univariate were included in the multivariable analyses (38).

### Prediction of the Molecular Subtypes and Various Gene Signatures in BLCA

There are several molecular subtype systems, including TCGA, CIT, Consensus, Lund, UNC, Baylor, and MDA subtypes (39–45). The molecular subtypes of patients were determined using ConsensusMIBC and BLCAsubtyping R packages. Then, we collected twelve bladder cancer signatures which were specific to different molecular subtypes (39, 45). After that, we collected more therapeutic signatures, including targeted therapy-associated gene signatures, oncogenic pathways associated with non-inflamed TME and gene signatures predicting radiotherapy responses for further investigation in BLCA therapies. The GSVA R package was used to calculate the enrichment scores of the signatures above (46).

### Cell Culture and Reagents

Human bladder cancer cell lines T24, 647-V were purchased from the Shanghai Institute of Cell Biology, Chinese Academy of Sciences. All cells were cultured with Dulbecco’s Modified Eagle’s Medium (DMEM; Gibco Company, Grand Island, NY, USA) with 10% fetal bovine serum (FBS; Gibco Company) and 1% penicillin/streptomycin (Invitrogen) and at 37°C with 5% CO2.

### Lentivirus Packaging and Plasmid Transfection

The target plasmids include as follows: sh-vector, sh*PMEPA1*-A, sh*PMEPA1*-B, oe-vector, and oe*PMEPA1*. According to its manufacturer, the above plasmids with the packaging plasmid psAX2 and envelope plasmid VSVG were transfected into 293T cells by lipofectamine 8000 (Invitrogen) instructions. The sh*PMEPA1*-A sequences were as follows: Sense: CCGGGAGTTTGTTCAGATCATCATCCTCGAGGATGATGATCTGAACAAACTCTTTTTG; anti-sense: AATTCAAAAAGAGTTTGTTCAGATCATCATCCTCGAGGATGATGATCTGAACAAACTC. The sh*PMEPA1*-B sequences were as follows: Sense: CCGGGTCCCTATGAATTGTACGTTTCTCGAGAAACGTACAATTCATAGGGACTTTTTG; anti-sense: AATTCAAAAAGTCCCTATGAATTGTACGTTTCTCGAGAAACGTACAATTCATAGGGAC. After 48 h, the virus was directly added to cells in a 6-well plate (or immediately frozen in -80°C freezer for future use) for 24 h, and after 48 h culture, we collected the cell protein to test the infection efficiency.

### Western Blot Analysis

Proteins were isolated from these transfected cells. Protein concentration was quantified by BCA protein assay. Using SDS-PAGE protein electrophoresis, the PVDF membranes were blocked with 5% non-fat milk in TBST, washed, and then probed overnight at 4°C with primary antibodies: Tubulin (T-5168, Sigma-Aldrich, 1:5000), *PMEPA1* (16521-1-AP, Proteintech, 1:1000). After washing, membranes were incubated with the suitable horseradish peroxidase-conjugated secondary antibody (Cell signaling, 1:5000) for 1 hour and re-washed 3 times. Then membranes were exposed using the ECL system (Thermo Fisher Scientific).

### Immunohistochemical Staining and Evaluation

After deparaffinization, rehydration, and antigen retrieval, TMA slides were blocked with 3% hydrogen peroxide and 5% BSA, washed and then probed overnight at 4°C with primary antibodies: *PMEPA1* (16521-1-AP, Proteintech, 1:200), CD68 (Kit-0026, Maxim). After washing, the slides were incubated with the suitable enhancer and secondary antibody (ZSGB-BIO, Beijing, China) for half an hour at room temperature. A DAB Substrate Kit was used for chromogenic reaction. Finally, the sections were counterstained with hematoxylin, then dehydrated, cleared and evaluated.

Immunostaining was evaluated under light microscopy at 200x magnification by two independent pathologists. PMEAP1 was observed in the membrane and/or cytoplasm of the BLCA cells. CD68 was observed in the membrane and/or cytoplasm of the macrophages. The H-score of *PMEPA1* in BLCA was calculated using software Inform 2.4.0. The absolute number of macrophages was counted manually. The total number of stained macrophages (in the central tumor and peritumoral stroma) was included in the analyses. Serial sections were used for the PMEPA1 and CD68 antibodies staining.

### Cell Proliferation and Colony Formation Assay

Two thousand cells were plated in 96-well plates, and 10 ul CCK8 solution (B34304, Bimake) was added to each well after 24, 48, 72, 96 and 120 hours. Then, the cells were incubated for 1 hour at 37 °C and 5% CO2. The supernatants were added to new 96-well plates, and the optical absorbance was measured at 450 nm.

Five hundred cells/group were plated into 6-well plates. Cells were cultured with 2.5 ml media at 37°C in an incubator. After eight days of cultivation, cells were gently washed by PBS, fixed by formalin, and stained by 0.1% crystal violet, and the cloning efficiency was determined.

### Wound Healing and Transwell Assay

Cells from each group were plated into 6-well plates at around 95% confluence. Then, we used a 200 ul pipette tip to make symmetrical wounds. After being washed by PBS twice, cells were incubated with a non-serum DMEM medium for 24 h (or 48 h). Migration pictures were taken at 0 h and 24 h (or 48 h) after drawing the wound. The wound distance of each group at 40x magnification was measured by Image J software. Each experiment was performed in triplicate.

Transwell migration and transwell invasion assays were conducted using 8-um transwell chambers (Corning Company, NY, USA). 2 x 104 cells were seeded into the upper chambers of 24-well with a non-serum medium, and 600 ul of 15% FBS medium was added into lower chambers. The matrigel (Corning Company, NY, USA) was plated in the upper chamber for the invasion assays, but not migration assays. Moreover, cells were incubated for 24 h in migration assays and 48 h in the invasion assays. The cells in the chamber were fixed by formalin and stained by 0.1% crystal violet after incubating. Invaded cells were counted in 3 random fields per well under a 100x microscope.

### Statistical Analysis

The SPSS statistical software (version 26.0.0), R software v4.0.3 and GraphPad 7.0 software were used for data analysis. Overall survival (OS) was defined as the time interval between surgery date and date of death. Pearson or Spearman coefficients were used to calculate the correlations between variables. Continuous variables fitting a normal distribution between two groups were compared using a t-test. Otherwise, the Mann-Whitney U test was conducted. Survival analyses were performed by Kaplan–Meier curves and p-values were calculated by the Log-rank test. All statistical tests were two-sided. P < 0.05 was considered statistically significant.

## Results

### Identification of *PMEPA1 via* Bioinformatical Analysis of the GEO and TCGA Databases

**Figure 1:**
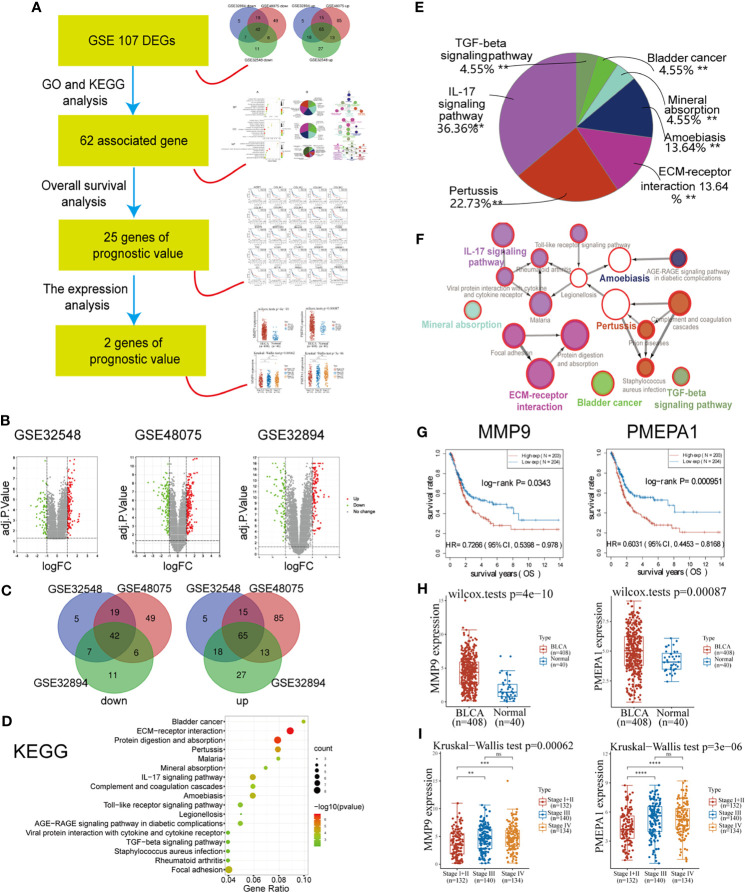
Identification of *PMEPA1 via* bioinformatical analysis of the GEO and TCGA databases. **(A)** The workflow of **Figure 1**. **(B)** Volcano plots were constructed using fold-change values and adjusted P. The red point in the plot indicates the over-expressed mRNAs, and the green point represents the down-expressed mRNAs with statistical significance (p < 0.05). **(C)** Authentication of 107 common DEGs in the three datasets (GSE32894, GSE48075, and GSE32548) through Venn diagrams software (available online: http://bioinformatics.psb.ugent.be/webtools/Venn/). Different colors indicate different datasets. **(D–F)** Kyoto Encyclopedia of Genes and Genomes (KEGG) pathway analysis of 107 common DEGs. **(D)** The bubble chart of the top 17 significant KEGG terms. **(E)** Pie graph of specific Cluster enriched from KEGG terms. **(F)** The KEGG regulation network of 107 genes. **(D–F)** were calculated using Cytoscape 3.6.1 and ClueGO v2.5.7. **(G)** The survival curve of MMP9 and *PMEPA1*. Raw counts of RNA-sequencing data (level 3) and corresponding clinical information from BLCA were obtained from the TCGA dataset. For Kaplan–Meier curves, p-values and hazard ratio (HR) with 95% confidence interval (CI) were generated by log-rank tests and univariate Cox proportional hazards regression. **(H)** The expression distribution of MMP9 and *PMEPA1* in BLCA tissues and normal tissues. All data of normal tissue samples were obtained from BLCA in the GTEx V8 release version (https://gtexportal.org/home/datasets). **(I)** The expression distribution of MMP9 and *PMEPA1* among different pTNM stages. All data of BLCA samples were obtained from TCGA dataset.

The workflow of this part of the work is shown in **Figure 1A**. There were 203 MIBC tissues and 372 NMIBC tissues in our present study. *Via* GEO2R online tools, we extracted 212, 340, and 211 DEGs from GSE32894, GSE48075, and GSE32548 between MIBC and NMIBC samples (**Table S2**–**S4** and **Figure 1B**), respectively. Then, Venn diagram software was used to identify the DEGs in common. Results showed that 107 DEGs were detected, including 42 down-regulated genes (logFC < -1) and 65 up-regulated genes (logFC > 1) (**Table S5** and **Figure 1C**).

The results of GO and KEGG analysis showed a strong association with cancer invasion and tumor microenvironment (TME). A total of 177 GO terms of biological process, 10 GO terms of cellular component, 19 GO terms of molecular function and 22 pathways of KEGG were identified to be significant (false discovery rate, or FDR < 0.05), (**Table S6**, **Figure 1D** and **Figure S1A**). To further investigate the potential functions of the DEGs, these GO terms above were enriched in several specific functional groups (**Table S7**, **Figures 1E** and **S1B**), and the regulation network constructions of these groups were calculated using ClueGO v2.5.7 employing medium network specificity (**Figures 1F** and **S1C**). Those functional groups strongly associated with cancer invasion and TME were listed as follows: for biological processes (BP), “extracellular matrix disassembly (10%)” and “extracellular structure organization (20%)”; for GO cell component (CC), “collagen-containing extracellular matrix (20%)” and “fibrillar collagen trimer (30%)”; for molecular function (MF), “extracellular matrix structural constituent conferring compression resistance (5.26%)” and “extracellular matrix structural constituent conferring tensile strength (15.79%)”; for KEGG, “ECM-receptor interaction (13.64%)” and “IL-17 signaling pathway (36.36%)”.

Considering the KEGG terms found above are strongly associated with cancer invasion and tumor microenvironment, those genes in 22 KEGG pathways could be the potential targeted gene. Then 62 genes of 107 DEGs were found associated with these 22 KEGG pathways (**Table S5**). After that, survival analysis indicated that 25 of 62 genes were found to have prognostic value (P < 0.05) (**Table S5**, **Figures 1G** and **S2**). At last, we investigated the expression of 25 genes between BLCA and normal tissue and found two up-regulated genes (*PMEPA1* and MMP9) with significantly high expression (P < 0.05) (**Table S5** and **Figure 1H**). The significant difference of the expression in different pTNM stages also indicated their prognostic significance in tumor progression (**Figure 1I**).

### Significant Pathways Influenced by *PMEPA1* in TCGA and GSE31684

**Figure 2:**
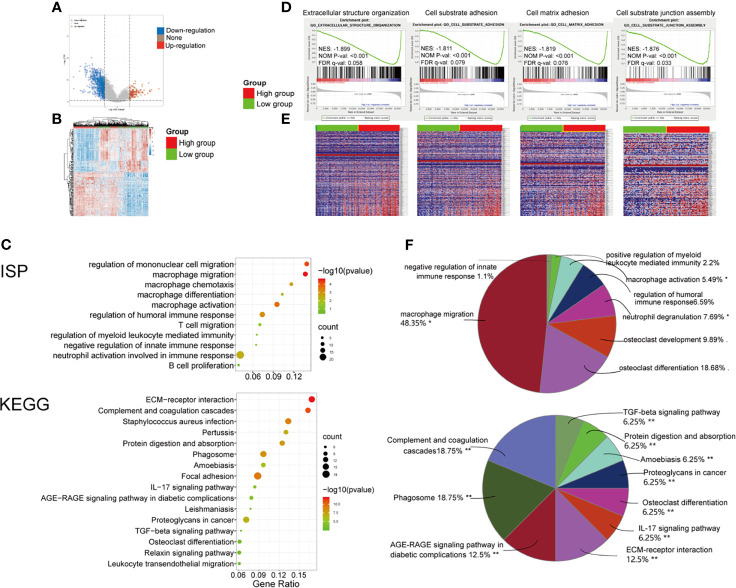
Significant pathways influenced by *PMEPA1* in TCGA and GSE31684. **(A, B)** Significant pathways influenced by *PMEPA1* in TCGA. **(A)** Volcano plots were constructed using fold-change values and adjusted P. The red point in the plot represents the over-expressed mRNAs, and the blue point indicates the down-expressed mRNAs with statistical significance. **(B)** Hierarchical clustering analysis of mRNAs, which were differentially expressed between high-*PMEPA1* expression tissues and low-*PMEPA1* expression tissues. **(C, F)** GO and KEGG analysis of top 200 up-regulated genes. **(C)** The top 10 significant GO terms for the immune system process were listed, and the top 16 significant KEGG terms were listed. **(F)** Pie graph of specific Cluster of GO and KEGG analysis. **(D, E)** Significant pathways influenced by *PMEPA1* in GSE31684 using GSEA analysis. **(D)** Profile of the running ES score and positions of gene set members on the rank-ordered list. GSEA, gene set enrichment analysis; ES, enrichment score; NES, normalized enrichment score; NOM p-value, normalized p-value; FDR q-value, false discovery rate q-value. **(E)** Blue-Red O’ Gram in the space of the analyzed genesets, Red color indicated high expression, and blue color indicated low expression. *P < 0.05; **P < 0.01.

To determine the function of the *PMEPA1* in BLCA, we applied the Limma package (version: 3.40.2) of R software to study the DEGs between low- and high-*PMEPA1* expression tissue in TCGA and 125 up-regulated DEGs and 804 down-regulated DEGs were found (**Table S8** and **Figures 2A, B**). Additionally, GO and KEGG analyses were performed to analyze the top 200 down-regulated genes, which indicated the potential function of *PMEPA1*. The results also showed a strong association with cancer invasion and TME, which included 48 GO terms of BP, 10 GO terms of CC, 16 GO terms of MF, 91 GO terms of Immune System Process (ISP) and 16 pathways of KEGG (**Table S9**, **Figures 2C** and **S3**). After specific cluster enrichment analysis of the GO terms above, 51 functional groups were determined (**Table S10** and **Figure 2F**). Those functional groups strongly associated with cancer invasion and TME were listed as follows: for GO BP, “cell adhesion mediated by integrin (2.08%)”, “cell-substrate junction assembly (4.17%)”, “collagen catabolic process (6.25%)”, “collagen fibril organization (2.08%)”, “extracellular matrix organization (4.17%)”, “macrophage migration (10.42%)” and “myeloid leukocyte migration (20.83%)”; for GO CC, “extracellular matrix (20%)” and “fibrillar collagen trimer (30%)”; for GO MF, “fibronectin binding (6.25%)”, “integrin binding (6.25%)”, “extracellular matrix structural constituent conferring compression resistance 6.25%)” and “extracellular matrix structural constituent conferring tensile strength (6.25%)”; for GO ISP, “macrophage activation (5.49%)”, “macrophage migration (48.35%)”, “negative regulation of innate immune response (1.1%)” and “positive regulation of myeloid leukocyte mediated immunity (2.2%)”; for KEGG, “ECM-receptor interaction (12.5%)”, “Complement and coagulation cascades (18.75%)”, “IL-17 signaling pathway (6.25%)” and “TGF-beta signaling pathway (6.25%)”.

Moreover, GSEA was conducted using 93 cases of subjects from the GSE31684 cohort that were classified into a high-*PMEPA1* (n=47) and a low-*PMEPA1* group (n=46). Gene sets were considered significantly enriched based on FDR q-value, NOM P-value and NES. The results showed that several canonical pathways that are involved in cancer invasion and TME, such as “extracellular structure organization”, “cell-substrate adhesion”, “cell-matrix adhesion”, and “cell-substrate junction assembly” were particularly enriched in the high-*PMEPA1* group. (**Figures 2D, E**).

These results demonstrate that the function of *PMEPA1* may be strongly associated with cancer invasion and TME, especially macrophage activation and migration.

### The Effect of *PMEPA1* on Immunological Status in Pan-Cancers Using the TISIDB

**Figure 3:**
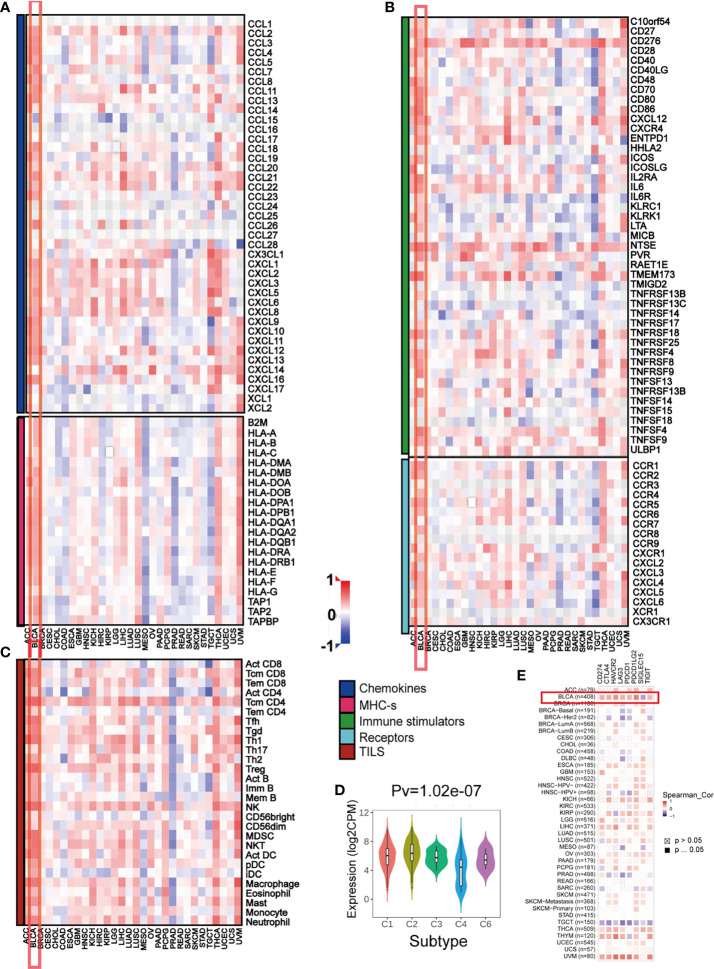
The effect of *PMEPA1* on immunological status in pan-cancers using the TISIDB. **(A, B)** Correlation between *PMEPA1* and 124 immunomodulators (chemokines, MHC-s, immune stimulators and receptors). **(C)** Correlation between *PMEPA1* and 28 tumor-associated immune cells. The color indicates the correlation coefficient. The horizontal axis represents different tumor tissues, and the vertical axis represents the expression of immunomodulators and the infiltration levels of immune cells. **(D)** *PMEPA1* mRNA expression in different immune subtypes in BLCA, C1 (would healing); C2 (IFN-gamma dominant); C3 (inflammatory); C4 (lymphocyte depleted); C5 (immunologically quiet); C6 (TGF-b dominant). **(E)** Correlation between *PMEPA1* and eight immune checkpoints, which were obtained from TIMER database. The horizontal axis represents the expression of immune checkpoint-related genes and the vertical axis represents different tumor tissues. Red color indicates positive correlation, blue color indicates negative correlation, and white color indicates no statistical significance.

Previous studies suggest the quantity and spatial distribution of stromal tumor-infiltrating lymphocytes (sTILs) within the tumor microenvironment (TME) predict stages of tumor inflammation and patient survival and correlate with the expression of immune checkpoints (47) Therefore, we tried to find whether *PMEPA1* expression was associated with immune infiltration in BLCA. Our findings revealed that *PMEPA1* was positively correlated with most immunomodulators (chemokines, MHC-s, immune stimulators and receptors) in BLCA (**Figures 3A, B**). Likewise, *PMEPA1* was positively correlated with the majority of TIICs in BLCA (**Figure 3C**). We then analyzed mRNA expression patterns of *PMEPA1* in different immune subtypes, which showed significant differences (**Figure 3D**). In the end, we proved that the expression of *PMEPA1* was mutually associated with several immune checkpoints, including PD-1 (PDCD1), PD-L1 (CD274), CTLA-4, HAVCR2 (TIM-3), PDCD1LG2, TIGIT and LAG-3 in BLCA (**Figure 3E** and **Table S11**).

In summary, the overexpression pattern of *PMEPA1* is TME specific, and the immunostimulating effect of *PMEPA1* in TME is the most obvious in BLCA.

### Correlation Analysis Between *PMEPA1* and the Infiltration Levels of Tumor-Infiltrating Immune Cells in TIMER

To further investigate the relationships between *PMEPA1* and the different immune cells, we calculated the infiltration levels of TIICs including TAMs, cancer associated fibroblasts (CAFs), myeloid-derived suppressor cells (MDSCs), monocytes, neutrophils, tregs, myeloid dendritic cells (DCs), NK cells, B cells, CD4+ T cells, and CD8+ T cells using eight independent algorithms in TIMER (**Table S12** and **Figure S4**). The results showed that *PMEPA1* was positively correlated with the infiltration levels of macrophages, CAFs, MDSCs, monocytes, and neutrophils in different algorithms, but mostly negatively correlated with the infiltration levels of CD8+ T cells, CD4+ T cells, and B cells.

Next, we investigated the correlation between *PMEPA1* expression and the status of tumor-infiltrating immune cells based on immune marker gene expression levels *via* the TIMER databases. The immune cells analyzed included CD8+ T cells, CD4+ T cells, B cells, tumor-associated macrophages (TAMs), monocytes, M1 and M2 macrophages, cancer-associated fibroblasts (CAFs), myeloid-derived suppressor cells (MDSCs), neutrophils, DCs, and natural killer (NK) cells. Moreover, different subsets of T cells, namely follicular helper T (Tfh), regulatory T (Tregs), T helper 1 (Th1), Th2, Th17, and exhausted T cells were also analyzed. Since tumor purity of clinical samples influences the immune infiltration analysis, the correlation analysis was adjusted for purity (**Table S11**). In line with the previous results, *PMEPA1* expression in BLCA tissues significantly correlated with marker genes expression from tumor-infiltrating monocytes, TAMs, DCs, Tregs, CAFs, MDSCs, and exhausted T cells.

In summary, the *PMEPA1* expression was strongly positively correlated with particular TIICs, including monocytes, TAMs, CAFs, and MDSCs in the TME. This suggests that *PMEPA1* plays an important role in regulating tumor immunity.

### The Effect of *PMEPA1* on the TME in Three Datasets (GSE32894, GSE48075, and GSE13507)

**Figure 4:**
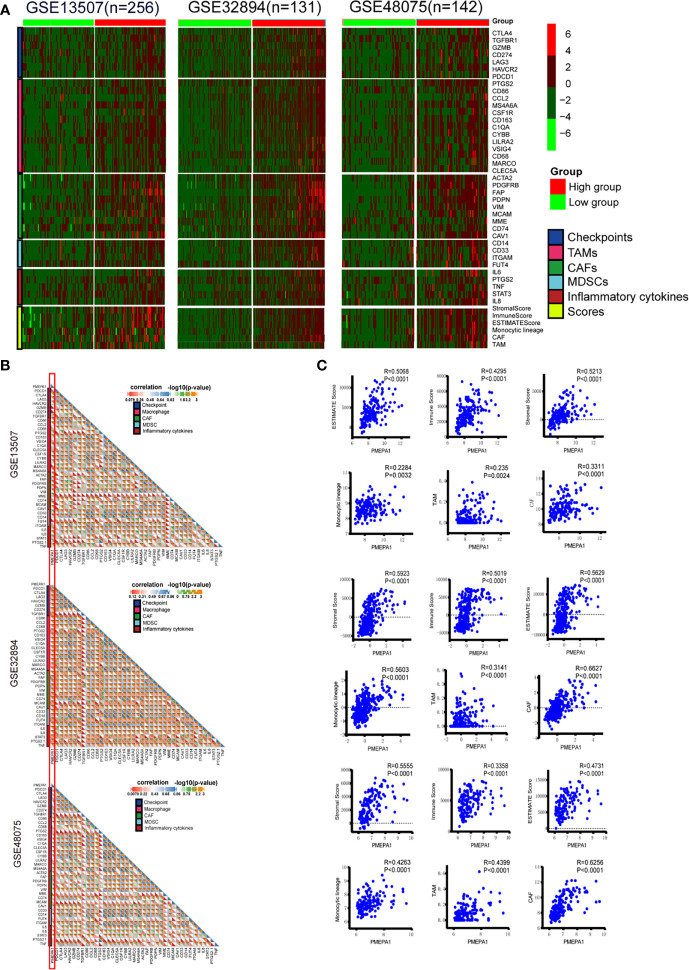
The effect of *PMEPA1* on TME in three datasets [GSE32894(n=131), GSE48075(n=142), GSE13507(n=256)]. **(A)** Differences in the expression of 38 immune genes (immune checkpoints, marker genes of TAMs, CAFs, MDSCs, and inflammatory cytokines), three immune scores (immune score, stromal score, and ESTIMATE score) of TME and the infiltration levels of three types of TIICs (TAMs, CAFs, and MDSCs) between high- and low-*PMEPA1* groups in BLCA. The red color indicates high expression of immune genes, and green color indicates low expression of immune genes. **(B)** Correlation between *PMEPA1* and 38 immune genes (immune checkpoints, marker genes of TAMs, CAFs and MDSCs and inflammatory cytokines). Every little square indicates the correlation between the genes in horizontal axis and vertical axis, the color and the value of the triangle on the lower left indicate the Pearson correlation coefficient, the color and the value of the triangle on the upper right indicate the statistical p-value. **(C)** The scatterplots display the Pearson’s rho value and p-value of the correlation between *PMEPA1* and three TIICs (TAMs, CAFs, and MDSCs) levels and three immune scores of TME.

To verify the findings above, we further investigated the effect of *PMEPA1* on the TME in three datasets (GSE32894, GSE48075, and GSE13507). Firstly, we applied the ESTIMATE R package to calculate immune and stromal scores, which predicted the infiltration of nontumor cells, and CIBERSORT, MCPcounter R package to calculate the infiltration scores of monocytic lineage, TAMs and cancer-associated fibroblasts (CAFs) (**Table S13**-**15**). The immune marker genes of checkpoints, TAMs, CAFs, MDSCs and inflammatory cytokines were identified from previous studies. Then we divided the patients of these datasets into high- and low-*PMEPA1* expression groups and evaluated the differences of genes of checkpoints, TAMs, CAFs, MDSCs, inflammatory cytokines and six scores between these two groups, which showed remarkably more enrichment in the high-*PMEPA1* group (**Figure 4A**). Moreover, the correlation analysis showed a significant positive association between *PMEPA1* and 38 immune genes (immune checkpoints, marker genes of TAMs, CAFs, MDSCs, and inflammatory cytokines), the infiltration levels of three TIICs (TAMs, CAFs, and MDSCs) and three immune scores of TME (**Figures 4B, C**). Considering the potent immunoregulatory properties of prostaglandins (PTGS2) and TGFbeta (TGFB1), we evaluate the expression of PMEPA1, TGFB1, and PTGS2 in tumor cells, TAMs, CAFs, endothelial cells, T-cells, muscle cells, and urothelial cells *via* single-cell mRNA sequencing data acquired from GSE145137 (48). The results show PMEPA1 were mainly expressed in basal tumor cells, CAFs, endothelial cells, muscle cells, and urothelial cells, TGFB1 were mainly expressed in basal tumor cells, CAFs, and TAMs, PTGS2 were hardly expressed in those cells (**Figure S5**). Moreover, we investigate the correlation between TGFB1, PTGS2, and PMEPA1 in TIMER, which show a remarkably positive association between TGFB1 and PMEPA1 (Partial.Cor = 0.453, P=5.27e-20) (**Figure S6**). To further investigate the function of PMEPA1 in BLCA, we evaluated the correlation between PMEPA1 and TGFB1, chemokines, and immune checkpoints in tumor cells, CAFs, and TAMs. The result shows that in tumor cells, PMEPA1 were negatively correlated with some chemokines (including CXCL1, CXCL11, CXCL2, CXCL8, CX3CL1) and positively correlated with CXCL14 and LAG3. In CAFs, PMEPA1 were negatively correlated with some chemokines (including CCL2, CCL19, CXCL12, CXCL14, CXCL2) and LAG3 and positively correlated with TGFB1. In TAMs, PMEPA1shows no significant correlation with these genes. At last, in all three cells, PMEPA1 were negatively correlated with most chemokines (including CCL2, CCL3, CCL4, CCL5, CCL8, CCL11, CCL13, CCL18, CCL19, CCL20, CCL23, CXCL1, CXCL10, CXCL12, CXCL16, CXCL2, CXCL3, CXCL8, CX3CL1), HAVCR2 and LAG3 and positively correlated with TGFB1, CXCL14, CD274, and SIGLEC15 (**Figure S7A**). Moreover, we evaluated the expression of genes that showed significant correlation with PMEPA1 in tumor cells, CAFs, and TAMs, which showed the PMEPA1 was mainly expressed in tumor cells and CAFs, TGFB1 was expressed in all three cells, and most of the chemokines were highly expressed in TAMs (**Figure S7B**). In conclusion, the *PMEPA1* expression was strongly positively correlated with the inflammation, infiltration levels of three TIICs (TAMs, CAFs, and MDSCs) and immune/stromal scores in TME.

### *PMEPA1* Promotes Bladder Cancer Cell Growth, Colony Formation Abilities, and Cell Migration and Invasion *In Vitro*

**Figure 5:**
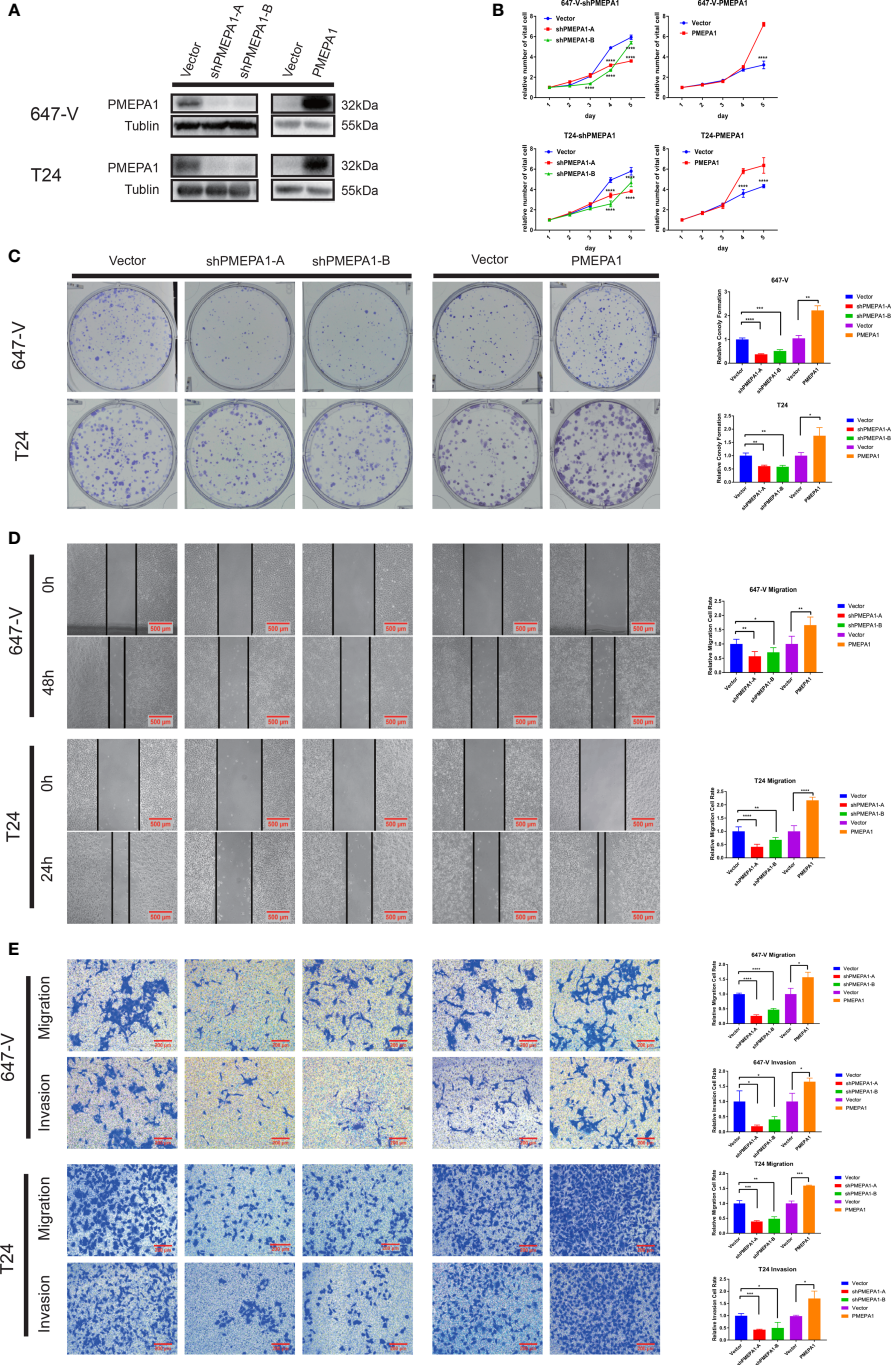
*PMEPA1* promotes bladder cancer cell growth, colony formation (CF) abilities, and cell migration and invasion *in vitro*. **(A)** The efficiency of *PMEPA1* knocked down and overexpressed has been testified by Western blot in T24 and 647-V. **(B)** The proliferation of T24 and 647-V cells with *PMEPA1* knocked down and overexpressed was detected by CCK8 assay (n = 3). **(C)** The proliferation of T24 and 647-V cells with *PMEPA1* knocked down and overexpressed was detected by plate clone assay (n = 3). **(D)** The cell migration of T24 and 647-V cells with *PMEPA1* knocked down and overexpressed was detected by wound healing assays (n = 3). **(E)** The cell migration and invasion of T24 and 647-V cells with *PMEPA1* knocked down and overexpressed was detected by transwell assays (n = 3). The * markers indicate the statistical analysis is between *PMEPA1* knocked down, overexpressed and vector. *P < 0.05; **P < 0.01; ***P < 0.001; ****P < 0.0001.

To investigate the role of *PMEPA1* in bladder cancer, firstly, we selected cell lines, T24 and 647-V for building the stable *PMEPA1* knockdown and overexpression cell lines. We then tested the protein level of *PMEPA1* by Western blot, and we investigated the influence on bladder cancer cells with the comparison of relative control cell lines (**Figure 5A**). To gain insight into the function of *PMEPA1* in BLCA progression, we utilized CCK8 assays to test cell viability after silencing *PMEPA1* expression in T24 and 647-V cell lines, and results revealed a significantly decreased cell growth (**Figure 5B**). Inversely, cell growth was significantly increased after *PMEPA1* overexpression in T24 and 647-V cell lines compared with the vector control group (**Figure 5B**). Furthermore, clones formation (CF) assay was applied to determine the *PMEPA1*’s CF ability function. Results showed that sh*PMEPA1* in T24 and 647-V cells significantly reduced CF ability (**Figure 5C**), and overexpression *PMEPA1* in T24 and 647-V increased CF ability (**Figure 5C**). Moreover, we applied wound healing and transwell assay to investigate the effects of *PMEPA1* expression on migration and invasion of BLCA cells. The results showed that cell migration and invasion abilities were significantly decreased after knocking down *PMEPA1* expression in T24 and 647-V cell lines. Inversely, overexpression of *PMEPA1* significantly increased these abilities (**Figures 5D, E**).

To summarize, *PMEPA1* promotes bladder cancer cell malignancy including cell growth, colony formation, cell migration, and invasion abilities.

### Preliminary Experimental Verification of *PMEPA1* Signature in BLCA

To verify the above results, we focused on *PMEPA1* expression, and its relationship with the clinical parameters and immune cells in BLCA. Also, we conducted an *in vitro* experiment using tissue samples collected from the Xiangya Hospital of Central South University.

**Figure 6:**
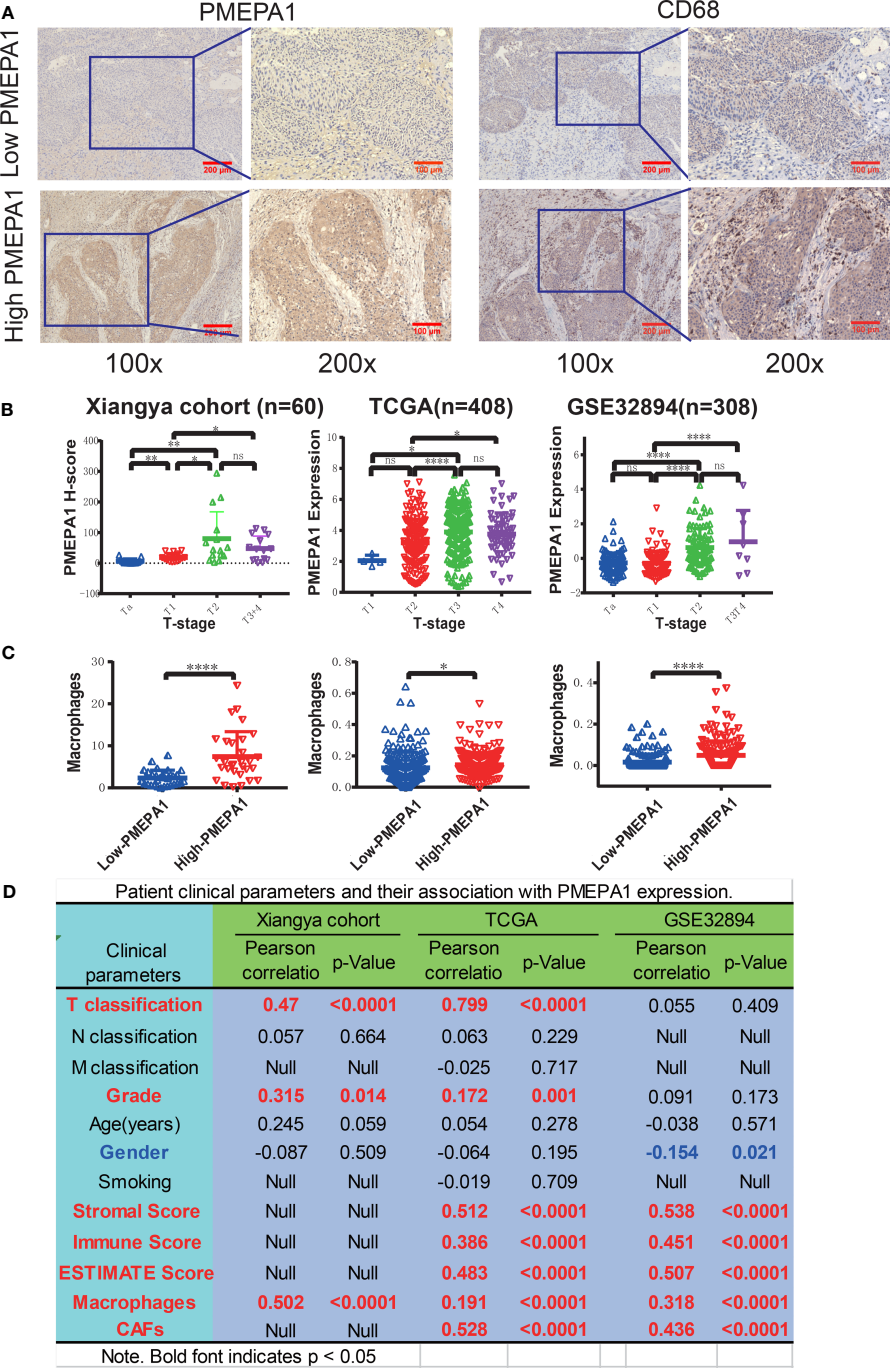
Preliminary experimental verification of *PMEPA1* signature in BLCA. **(A)** Immunohistochemical staining of *PMEPA1* and macrophages (CD68) in the low- and high-*PMEPA1* group at 100X and 200X magnification. Serial sections were used for the two antibody staining. **(B)** The expression of *PMEPA1* in BLCA tissues of different T-stage in three datasets [Xiangya cohort (n=60), TCGA (n=408), GSE32894 (n=308)]. The horizontal axis represents different T-stages, and the vertical axis represents the expression level of PMEPA1, **(C)** The level of TAMs infiltration investigated for the low- and high-*PMEPA1* group in three datasets above. The vertical axis represents the infiltration level of TAMs, **(D)** The patient clinical parameters and their association with *PMEPA1* expression in three datasets above. The asterisks indicated a statistically significant p-value (*P < 0.05; **P < 0.01; ****P < 0.0001; ns, P ≧ 0.05).

Firstly, we performed IHC to evaluate the expression and prognostic value of *PMEPA1* in a cohort of 60 BLCA specimens (**Table S1**), and the expression of *PMEPA1* and the clinical information of TCGA and GSE32894 were downloaded from TCGA and GEO (**Table S16**, **S17**). All of the patients were divided into low- and high-*PMEPA1* groups. Images of representative low- and high-*PMEPA1* samples were taken at 100x and 200x magnification (**Figure 6A**). The result showed that the TAMs, CAFs and three calculated scores (stromal score, immune score, and ESTIMATE score) were strongly associated with *PMEPA1* expression in three datasets (Xiangya cohort, TCGA, GSE32894) (**Figures 6A–D**). Moreover, the *PMEPA1* expression was positively correlated to tumor T-classification and grade in two datasets (Xiangya cohort and TCGA), which indicated the *PMEPA1* can predict the progression of BLCA (**Figures 6B, D**).

### Survival Analysis of the Clinical Parameters and *PMEPA1* of the Three Datasets Above

**Figure 7:**
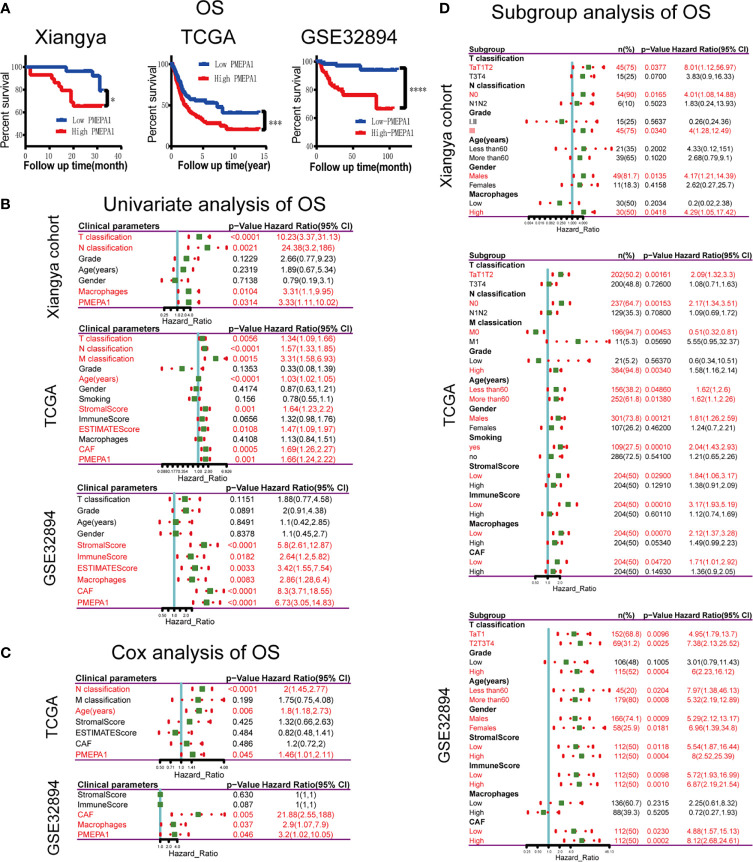
Survival analysis of clinical parameters and *PMEPA1* in three datasets above. **(A)** Kaplan-Meier survival curve analysis of the low- and high-*PMEPA1* group in three datasets [Xiangya cohort (n=60), TCGA (n=408), GSE32894 (n=308)]. **(B)** Univariate survival analysis of clinical parameters and *PMEPA1* expression with OS in three datasets above. **(C)** Cox survival analysis of clinical parameters and *PMEPA1* expression with OS in TCGA and GSE32894. **(D)** Univariate survival analysis of *PMEPA1* expression in subgroups with different clinical parameters. The asterisks indicated a statistically significant p-value (*P < 0.05; ****P < 0.0001). OS, overall survival.

The survival curves revealed that patients with high expression of *PMEPA1* had an unfavorable overall survival (OS) (**Figure 7A**). The significant risk factors of OS found by univariate survival analysis in the three datasets were listed below: T-classification, N-classification, macrophages, and *PMEPA1* in Xiangya cohort; T-classification, N-classification, M-classification, age, stromal score, ESTIMATE Score, CAFs and *PMEPA1* in TCGA; stromal score, immune score, ESTIMATE Score, macrophages, CAFs and *PMEPA1* in GSE32894 (**Figure 7B**). Furthermore, significant factors (with p < 0.05) above were included in the multivariable cox analysis, which showed that N-classification (p < 0.0001, HR=2, 95%CI=1.45-2.77), age (p=0.006, HR=1.8, 95%CI=1.18-2.73), *PMEPA1* (p=0.045, HR=1.46, 95%C=1.01-2.11) in TCGA; and CAFs (p=0.005, HR=21.88, 95%CI=2.55-188), macrophages (p=0.037, HR=2.9, 95%CI=1.07-7.9) and *PMEPA1* (p=0.046, HR=3.2, 95%CI=1.02-10.05) in GSE32894 were independent prognostic predictors (**Figure 7C**).

Additionally, we investigated the prognostic value of *PMEPA1* in selective patient subgroups of BLCA classified by clinicopathological factors (**Figure 7D**). In Xiangya cohort, *PMEPA1* correlated with poor prognosis for OS in patients with lower T classification (Ta+T1+T2), high grade (III), male patients, high macrophages infiltration level and without lymph node metastasis (N0). In TCGA, *PMEPA1* correlated to poor prognosis in patients with lower T classification (Ta+T1+T2), lower M classification (M0), high grade (III), smoking, male patients, low immune infiltration levels of TME, and without lymph node metastasis (N0). Moreover, in GSE32894, high *PMEPA1* only showed greater significant prognostic value in patients with high grade (III).

These results demonstrate that *PMEPA1* was an independent prognostic predictor of OS, and its prognostic significance in BLCA patients was based on their clinical characteristics (especially in early-stage, high grade, and male patients) and the immune infiltration levels of TME.

### *PMEPA1* Predicts the Molecular Subtype and the Therapeutic Response to Several Therapies in BLCA

**Figure 8:**
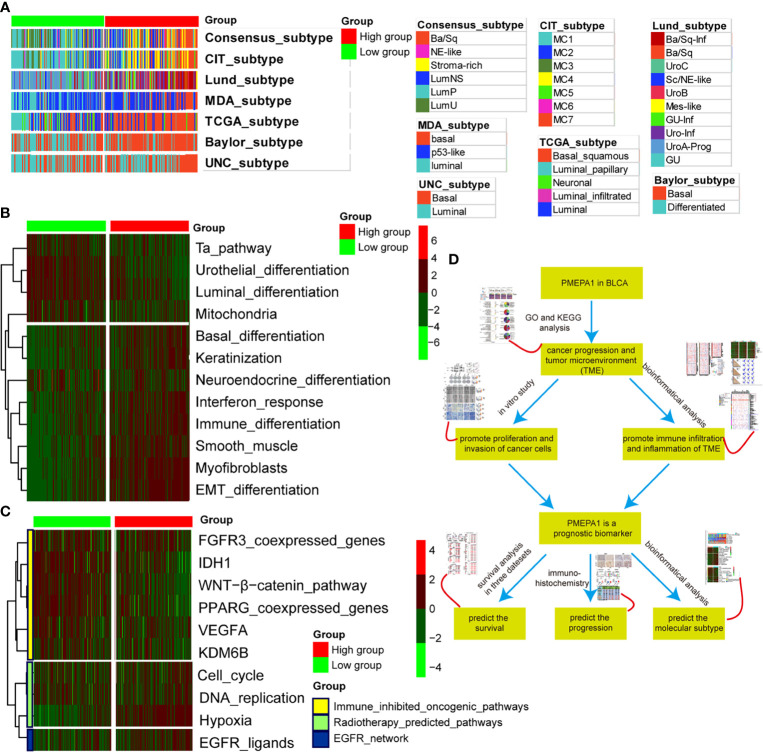
*PMEPA1* predicts the molecular subtype and the therapeutic response to several therapies in BLCA. **(A)** Correlations between *PMEPA1* and molecular subtypes using seven different algorithms. **(B)** Correlations between *PMEPA1* and bladder cancer signatures. **(C)** Correlations between *PMEPA1* and the enrichment scores of several therapeutic signatures such as targeted therapy and radiotherapy. **(D)** The workflow of the rest of the work.

Previous studies had elucidated that basal-type BLCA showed a poor prognosis and the highest immune cell infiltration (41, 45, 49). BLCA with high *PMEPA1* expression was more likely to be the basal subtype among the seven molecular subtyping systems (**Figure 8A**). This re-validated the conclusion that *PMEPA1* can predict prognosis based on immune infiltration levels of TME. In addition, the enrichment scores for the Ta pathway, urothelial differentiation, luminal differentiation and mitochondria were lower in the high-*PMEPA1* group. But the enrichment scores for basal differentiation, keratinization, interferon response, immune differentiation, smooth muscle, myofibroblasts and EMT differentiation were greater in the high-*PMEPA1* group (**Figure 8B**).

Moreover, a molecular subtype can also predict the clinical response to immunotherapies (anti-PD1/PD-L1 therapies), neoadjuvant chemotherapy, radiotherapy and several targeted therapies (39, 45, 50). Basal subtype tumors were more likely to respond to immunotherapies and neoadjuvant chemotherapy. Additionally, the calculated scores for EGFR ligands and radiotherapy-predicted pathways were higher in the high-*PMEPA1* group (**Figure 8C**). This shows that immunotherapies (anti-PD1/PD-L1 therapies), neoadjuvant chemotherapy, radiotherapy and ERBB therapy can be used, either alone or in combination, to treat BLCA with high *PMEPA1* expression. BLCA with lower *PMEPA1* expression was more likely to be the luminal subtype (**Figure 8A**). Immunotherapies (anti-PD1/PD-L1 therapies), radiotherapy and neoadjuvant chemotherapy were all unsuitable for BLCA with low *PMEPA1* expression. The enrichment scores for several immunosuppressive oncogenic pathways were significantly lower in the high-*PMEPA1* group (**Figure 8C**). Consistent with our previous conclusion, these oncogenic pathways were related to the non-inflamed TME in BLCA, which meant that the inflammation level of TME in the high-*PMEPA1* group was higher than the low-*PMEPA1* group,. The workflow of the rest of the work is shown in **Figure 8D**.

In conclusion, BLCA with high *PMEPA1* expression was more likely to be the basal subtype, which showed poor prognosis with more immune infiltration and a higher inflammation level of TME. Additionally, immunotherapies (anti-PD1/PD-L1 therapies), neoadjuvant chemotherapy, radiotherapy, and ERBB therapy were all suitable for treating BLCA with high *PMEPA1* expression.

## Discussion

Bladder cancer is one of the most common malignancies among urinary system tumors, with about 380,000 new cases and 150,000 deaths per year (1). It often recurs and progresses, especially in MIBC, bringing a great burden to society (51). However, there is little specific and efficient targeted therapy until now. Therefore, finding potential biomarkers of this cancer type and identifying its underlying mechanisms may help us find novel therapeutics for fighting against it.

To identify more useful prognostic biomarkers in bladder cancer, this study used bioinformatical methods based on three profile datasets (GSE32894, GSE48075, and GSE32548). 203 MIBC tissues and 372 NMIBC tissues were enrolled in the present research. *Via* GEO2R and Venn software, we revealed a total of 107 commonly changed DEGs (|logFC| > 1 and adjustPvalue < 0.05) comparing MIBC with NMIBC, including 42 down-regulated (Log FC < 0) and 65 up-regulated (Log FC > 0) DEGs. Then, the results of GO and KEGG analyses showed a strong association with cancer invasion and tumor microenvironment (TME) (**Figure 1**). This is consistent with previous reports that the tumor microenvironment is associated with tumor progression and metastasis (16, 17), and the results indicated that the 62 DEGs associated with the enriched GO terms may be potential biomarkers predicting tumor progression.

Therefore, we performed an overall survival analysis of these 62 genes and identified that 25 were found to have prognostic value (P < 0.05) (**Table S5** and **Figure S2**). Moreover, we investigated the expression of 25 genes between BLCA and normal tissues and found two up-regulated genes (*PMEPA1* and MMP9) with significantly high expression (**Table S5** and **Figure 1G**). Finally, it has been reported that MMP9 is involved in BLCA pathogenesis, and that it is significant in predicting overall survival.

*PMEPA1* is a multifunctional protein and has a growth-promoting or inhibitory role in prostate cancer, depending on whether the cancer cells are negative or positive to AR (5, 6). It can be up-regulated by transforming growth factor (TGF) while inhibiting TGF signaling through a negative feedback loop. In addition, the *PMEPA1* gene has been shown to induce tumorigenesis *via* interfering with other signaling cascades such as mutated p53, Hippo signaling, Wnt and EGF (9). However, *PMEPA1* has not previously been linked with BLCA prognosis and could serve as potential biomarkers for BLCA. To determine the function of the *PMEPA1* in BLCA, we applied the Limma package (version: 3.40.2) of R software to study the DEGs between low- and high-*PMEPA1* expression tissue in TCGA, and top 200 DEGs positively correlated with *PMEPA1* were analyzed by GO and KEGG analysis. The results also showed a strong association with cancer invasion and TME, especially macrophage migration and activation. Moreover, GSEA analysis was conducted using 93 cases of subjects from the GSE31684 cohort that were classified into a high-*PMEPA1* and a low-*PMEPA1* group, which reached a similar conclusion.

Increasing evidence suggests that the tumor microenvironment (TME) plays an important role in tumor progression and metastasis (16). The tumor microenvironment is composed of cancer cells and various types of nonmalignant cells such as myeloid-derived suppressor cells (MDSC), tumor-associated macrophages (TAMs), cancer-associated fibroblasts (CAFs) and immune cells. Previous studies suggest that the infiltration of stromal tumor-infiltrating lymphocytes (sTILs) in the TME predict stages of tumor inflammation and patient survival and correlate with the expression of immune checkpoints (47). Therefore, we tried to find whether *PMEPA1* expression was associated with immune infiltration in BLCA. Our findings revealed that *PMEPA1* was positively correlated with most immunomodulators (chemokines, MHC-s, immune stimulators and receptors) in BLCA (**Figures 3A, B**). Likewise, *PMEPA1* was positively correlated with the majority of tumor-infiltrating immune cells (TIICs) in BLCA (**Figure 3C**). Moreover, we proved that the expression of *PMEPA1* was mutually associated with several immune checkpoints, including PD-1 (PDCD1), PD-L1 (CD274), CTLA-4, HAVCR2 (TIM-3), PDCD1LG2, TIGIT, and LAG-3 in BLCA (**Figure 3E** and **Table S11**). Previous studies have shown that PD-L1 directly interacts with PD-1 to inhibit tumor cell apoptosis and negatively affect peripheral T effector cells (52, 53). Moreover, CTLA-4 is commonly regarded as a “brake” triggered by APC to activate CD4 + and CD8 + T cells (54), while TIM-3 has both negative and positive regulator functions. A combination of anti-CTLA4 and TIM-3 blockade was found to shrink the tumor in preclinical studies (55, 56). These correlations indicate a possible mechanism of *PMEPA1* regulation on immune infiltration in BLCA TME. *PMEPA1* expression, therefore, has a prognostic value in BLCA.

To further investigate the detailed relationships between *PMEPA1* and different immune cells, we calculated the infiltration levels of 11 types of TIICs using eight independent algorithms in TIMER (**Table S12** and **Figure S4**). The results showed *PMEPA1* was strongly positively correlated with the infiltration levels of macrophages, CAFs, MDSCs, monocytes, and neutrophils in different algorithms, but negatively correlated with the infiltration levels of CD8+ T cells, CD4+ T cells and B cells. Moreover, the correlation analysis between *PMEPA1* expression and TIICs based on immune marker genes expression levels showed a significant correlation with the monocytes, TAMs, DCs, Tregs, CAFs, MDSCs, and exhausted T cells (**Table S11**). All in all, the *PMEPA1* expression was strongly positively correlated with particular TIICs including monocytes, TAMs, CAFs, and MDSCs. This suggests that *PMEPA1* plays an important role in regulating tumor immunity, and therefore influences BLCA prognosis.

Among those nontumor cells, stromal cells and Immune cells take a critical place in the whole process of tumors from genesis to transferring and have a certain value in clinical diagnosis and prognosis. To verify the finding above, we further investigated the effect of *PMEPA1* on TME in three datasets (GSE32894, GSE48075, and GSE13507). Both heatmaps and correlation analysis showed a remarkably positive association between *PMEPA1* and 38 immune genes (immune checkpoints, marker genes of TAMs, CAFs, MDSCs, and inflammatory cytokines), the infiltration levels of three TIICs (TAMs, CAFs, and MDSCs) and three immune scores of TME (**Figure 4**).

As noticed by many research teams, prostaglandins and TGFbeta have potent immunoregulatory properties (57). We suppose that could be the possible mechanisms of the significant effect of *PMEPA1* on TME. The results of single-cell mRNA sequencing show PMEPA1 were mainly expressed in basal tumor cells and CAFs, TGFB1 were mainly expressed in basal tumor cells, CAFs and TAMs, PTGS2 were hardly expressed in those cells. In consideration of the remarkably positive association between TGFB1 and PMEPA1, we could infer that the TGFbeta produced by the tumor cells, CAFs, and TAMs could induce PMEPA1 in tumor cells and CAFs and at the same time attract macrophages and inhibit T cells. Moreover, the correlation analysis showed PMEPA1 were most strongly negatively correlated with chemokines in all three cells, and secondly correlated with part of chemokines in tumor cells and CAFs. However, PMEPA1shows no significant correlation with these genes in TAMs. Even more interesting is that PMEPA1 were positively correlated with TGFB1 just in CAFs (**Figure S7A**). And the expression analysis showed the PMEPA1 was mainly expressed in tumor cells and CAFs, TGFB1 was expressed in all three cells, and most of the chemokines were highly expressed in TAMs (**Figure S7B**). This provided further verification for the argument that, TGFbeta produced by CAFs, could induce PMEPA1 in tumor cells and CAFs, at the same time inhibit the expression of chemokine in tumor cells, CAFs and TAMs which thus inhibit the tumor immunity. Moreover the TAMs might be the most important effect cell and the TGFbeta produced by TAMs could form a positive feedback loop. Due to a tolerogenic cytokine milieu in the TME, recruited myeloid cells differentiate into immunosuppressive TAMs and MDSCs. TAMs are widely considered one of the main players in the regulation of the immune responses and are known to contribute to metastasis by priming the pre-metastatic site and promoting tumor cell extravasation and survival. Additionally, extensive TAMs infiltration has been shown to be positively correlated with cancer progression and poor clinical prognosis in various human cancers (58, 59). High numbers of peripheral blood MDSCs were found to adversely correlate with stage, grade and prognosis in bladder cancer. The MDSCs present in bladder tumors have been shown to express high levels of immunosuppressive molecules such as Arginase 1, inducible nitric oxide synthases (iNOS) and PD-L1 and directly suppress T-cell proliferation reflecting their phenotype in the peripheral blood (34). In the clinical setting, an increased number of MDSCs correlates with weakened clinical responses to immunotherapy (33). Moreover, low levels of circulating or tumor-infiltrating MDSCs have been attributed to an improved prognostic and predictive value in a variety of oncologic settings (36). Cancer-associated fibroblasts (CAFs, also known as myofibroblasts) are another major component in the tumor stroma. Previous studies show that CAFs play a vital role in establishing a metastatic niche and driving tumor cell proliferation, invasion and metastasis by secreting chemokines and cytokines in the microenvironment (32, 60, 61). Therefore, the significant effect of *PMEPA1* on TME may be one of the underlying mechanisms of predicting the progression and poor prognosis of patients.

Furthermore, our *in vitro* study demonstrated that silencing *PMEPA1* significantly decreased cell proliferation, migration and invasion (**Figure 5**). Inversely, when overexpressing *PMEPA1*, cell proliferation, migration and invasion were significantly increased, which may be another underlying mechanism of predicting the progression and poor prognosis of patients.

To verify the *PMEPA1* signature in BLCA, we focused on *PMEPA1* expression, relationship with clinical parameters and immune cells in BLCA, and the IHC result in the Xiangya cohort showed that the macrophages (in the central tumor and peritumoral stroma) were strongly associated with *PMEPA1* expression. Furthermore, the *PMEPA1* expression was significantly correlated to tumor T-classification and grade in two datasets (Xiangya cohort and TCGA), which indicated the *PMEPA1* may predict the progression of BLCA (**Figure 6**)

Next, survival analysis in three datasets (Xiangya cohort, TCGA, and GSE32894) showed significant risk factors including T-classification, N-classification, macrophages, and *PMEPA1* in Xiangya cohort; T-classification, N-classification, M-classification, age, stromal score, ESTIMATE Score, CAFs, and *PMEPA1* in TCGA; stromal score, immune score, ESTIMATE Score, macrophages, CAFs, and *PMEPA1* in GSE32894. Furthermore, multivariable cox analysis showed that N-classification, age, and *PMEPA1* in TCGA, and CAFs, macrophages, and *PMEPA1* in GSE32894 were independent prognostic predictors (**Figure 7C**). Finally, subgroup survival analysis suggested the prognostic significance of *PMEPA1* based on the clinical characteristics (especially in early stage, high grade, and male patients) and the immune infiltration level of TME.

Previous studies had elucidated that basal-type BLCA showed the highest immune cell infiltration and poor prognosis (41, 45, 49). BLCA with high *PMEPA1* expression was more likely to be the basal subtype among the seven molecular subtyping systems (**Figure 8A**). This re-validated the conclusion that *PMEPA1* can predict prognosis based on immune infiltration level of TME. Moreover, we show that immunotherapies (anti-PD1/PD-L1 therapies), neoadjuvant chemotherapy, radiotherapy, and ERBB therapy can be used, either alone or in combination, to treat BLCA with high *PMEPA1* expression.

This study had some limitations. First, the number of patients in our validation cohort was limited to 60 patients, and our results should be validated in larger sample sizes. Second, we did not determine the optimal cut-off value of *PMEPA1*. Here, the median *PMEPA1* mRNA expression was considered as the cut-off value. Third, algorithm analysis, based on RNA-seq, might not be sufficiently accurate. Finally, this calls for further experiments using vivo models to explore the potential biological mechanisms of *PMEPA1* in malignancy and tumor microenvironment (TME) of BLCA.

## Conclusion

We found that *PMEPA1* may be a novel potential biomarker in predicting the progression, prognosis, and molecular subtype of BLCA. We also provided an underlying mechanism by which *PMEPA1* expression might modulate the malignancy of cancer cells and the inflammation and immune infiltration levels of TME.

## Data Availability Statement

The datasets presented in this study can be found in online repositories. The names of the repository/repositories and accession number(s) can be found in the article/**Supplementary Material**.

## Ethics Statement

The studies involving human participants were reviewed and approved by Ethics Committee of the Xiangya Hospital of Central South University. The patients/participants provided their written informed consent to participate in this study.

## Author Contributions

DQ, JianH, JiaoH, and AY analysed the data has shown in **Figures 1**, **2**, **S1**, **S2**, and **Tables S1**, **S2**. TH and JD have contributed in **Figures 2**–**5**. XC, WR, XT, and QY have contributed in **Figures 6**–**8**, **S3**, and **S4**. BO, JC, and XZ have contributed in the manuscript writing. All authors have participated in editing and reviewed the manuscript.

## Funding

This work was supported by the National Natural Science Foundation of China (81873626, 81902592), Hunan Natural Science Foundation (2020JJ5884, 2018JJ2623), Hunan Province Key R&D Program (2019SK2202) and Xiangya Hospital Youth Fund (2018Q09).

## Conflict of Interest

The authors declare that the research was conducted in the absence of any commercial or financial relationships that could be construed as a potential conflict of interest.

## Publisher’s Note

All claims expressed in this article are solely those of the authors and do not necessarily represent those of their affiliated organizations, or those of the publisher, the editors and the reviewers. Any product that may be evaluated in this article, or claim that may be made by its manufacturer, is not guaranteed or endorsed by the publisher.

## Supplementary Material

Supplementary Figure 1 Gene Ontology (GO) including biological process, cellular component, and molecular.pdf. Function analysis of 107 common DEGs. **(A)** The bubble chart of top 10 significant GO terms for biological processes, cellular components, and molecular functions. **(B)** Pie graph of specific Cluster. **(C)** The GO regulation network of 107 genes. The enriched GO terms were calculated using Cytoscape 3.6.1 and ClueGO v2.5.7.

Supplementary Figure 2 The survival curve of the 23 associated genes in the TCGA set.pdf. R software version v4.0.3 was used to identify the prognostic information of the 62 associated genes and 25 of 62 genes had a significantly better or worse survival rate (P< 0.05)

Supplementary Figure 3 Significant pathways influenced by *PMEPA1* in TCGA.pdf. **(A, B)** Go and analysis of top 200 up-regulated genes. **(A)** The top 10 significant GO terms were listed for biological processes, cellular components, molecular functions. **(B)** Pie graph of specific Cluster.

Supplementary Figure 4 Correlation between *PMEPA1* and the infiltration levels of 11 types of TIICs (TAMs, cancer associated fibroblasts (CAFs), myeloid-derived suppressor cells (MDSCs), monocytes, neutrophils, tregs, myeloid dendritic cells (DCs), NK cells, B cells, CD4+ T cells, CD8+ T cells) in pan-cancers using the TIMER database.pdf. The horizontal axis represents different tumor tissues, the vertical axis represents different TIICs using 8 independent algorithms, different colors represent correlation coefficients, and negative values represent negative correlations. A positive value represents a positive correlation. The stronger the correlation, the darker the color. P value of < 0.05 is considered statistically significant.

Supplementary Figure 5 The expression of TGFB1, PTGS2 and PMEPA1 in tumor cells, TAMs, CAFs, endothelial cells, T-cells, muscle cells, urothelial cells. The horizontal axis represents different cell types, and the vertical axis represents the expression level of PMEPA1, which were plotted *via* Seurat package of R software. All single-cell mRNA sequencing data was acquired from GSE145137.

Supplementary Figure 6 Correlation analysis between TGFB1, PTGS2, and PMEPA1 in TIMER. The horizontal axis represents the expression level of PMEPA1 and the vertical axis represents the expression level of TGFB1 and PTGS2, The Partial.Cor and P-value were calculated after purity. Partial.Cor practical correlation coefficient.

Supplementary Figure 7 The correlation and expression of PMEPA1, TGFB1, chemokines, and immune checkpoints in tumor cells (n=36), CAFs (n=273) and TAMs (n=283). (A) The correlation between PMEPA1 and TGFB1, chemokines and immune checkpoints in tumor cells, CAFs and TAMs. The horizontal axis represents the correlation coefficient and the vertical axis represents the different genes, the P-value is listed on the right. P value of < 0.05 is considered statistically significant. (B) The expression of PMEPA1, TGFB1, chemokines, and immune checkpoints in tumor cells, CAFs and TAMs. The horizontal axis represents different genes in tumor cells, CAFs and TAMs and the vertical axis represents the expression level, All single-cell mRNA sequencing data was acquired from GSE145137.

Supplementary Figure 8 The expression of PMEPA1 and TGFB1 in mutated/no mutation groups of SMAD4 and TGFBR. The horizontal axis represents the mutation states of SMAD4 and TGFBR and the vertical axis represents the expression levels of PMEPA1 and TGFB1, All the data was acquired from cBIoportal website (http://www.cbioportal.org).

## Abbreviations

AR, androgen receptor; BLCA, bladder cancer; BP, biological processes; CAFs, cancer associated fibroblasts; CC, cell component; CF, colony formation; DEGs, differentially expressed genes; DC, myeloid dendritic cell; FDR q-value, false discovery rate q-value; GEO, Gene Expression Omnibus; GO, Gene Ontology; GSEA, gene set enrichment analysis; IHC, immunohistochemical; ISP, immune system process; KEGG, Kyoto Encyclopedia of Genes and Genomes; MDSCs, myeloid-derived suppressor cells; MM, multiple myeloma; MF, molecular function; MIBC, muscle-invasive bladder cancer; NMIBC, non-muscle-invasive bladder cancer; NOM P-value, normalized p-value; NES normalized enrichment score; NK, cell natural killer cell; OS overall survival; *PMEPA1*, prostate transmembrane protein androgen induced 1; STILs, stromal tumor-infiltrating lymphocytes; TCGA, The Cancer Genome Atlas; TME, tumor microenvironment; TIICs, tumor-infiltrating immune cells; TAMs, tumor-associated macrophages; TGF, transforming growth factor.
