# Supplementary figures and images for: PMEPA1 Is a Prognostic Biomarker That Correlates With Cell Malignancy and the Tumor Microenvironment in Bladder Cancer
Source: Front Immunol. 2021 Oct 28;12:705086. doi: 10.3389/fimmu.2021.705086 (PMC8582246; doi:10.3389/fimmu.2021.705086)

A

BP

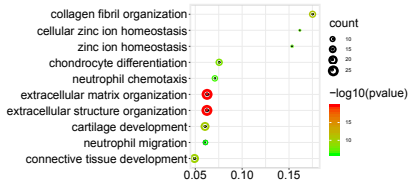

CC

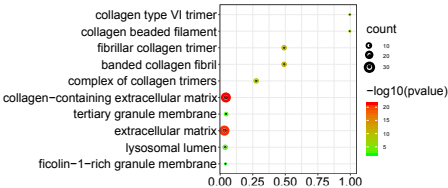

MF

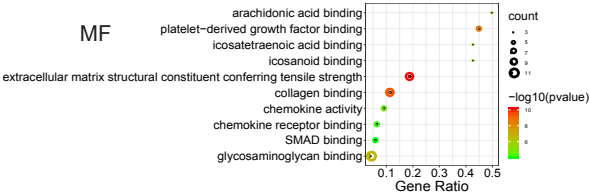

B

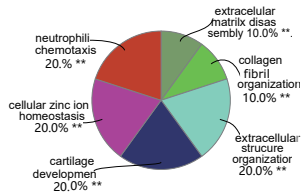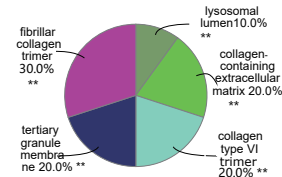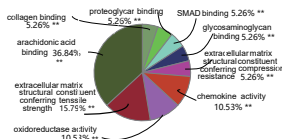

C

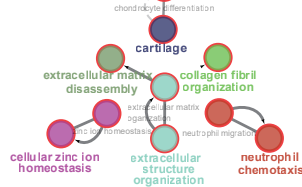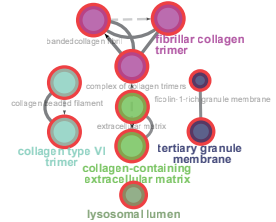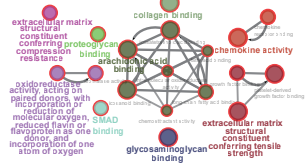

Supplement: Supplementary Figure 1 — Gene Ontology (GO) including biological process, cellular component, and molecular.pdf. Function analysis of 107 common DEGs. (A) The bubble chart of top 10 significant GO terms for biological processes, cellular components, and molecular functions. (B) Pie graph of specific Cluster. (C) The GO regulation network of 107 genes. The enriched GO terms were calculated using Cytoscape 3.6.1 and ClueGO v2.5.7. [file Image_1.pdf]

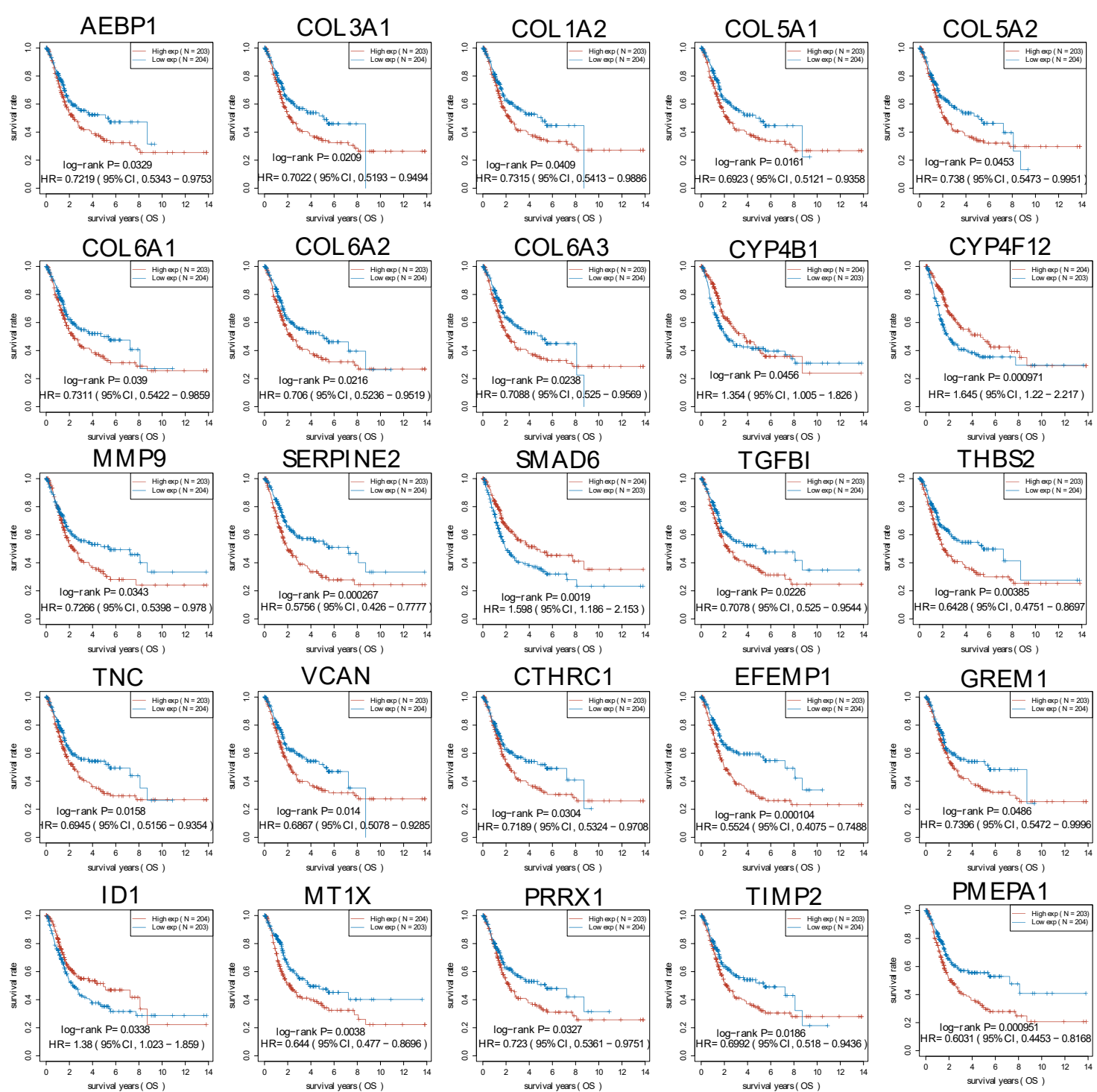

Supplement: Supplementary Figure 2 — The survival curve of the 23 associated genes in the TCGA set.pdf. R software version v4.0.3 was used to identify the prognostic information of the 62 associated genes and 25 of 62 genes had a significantly better or worse survival rate (P< 0.05) [file Image_2.pdf]

A

BP

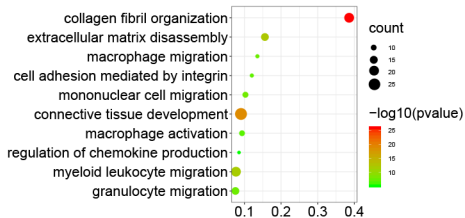

B

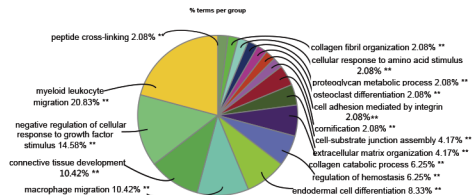

CC

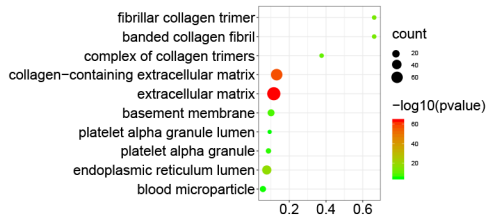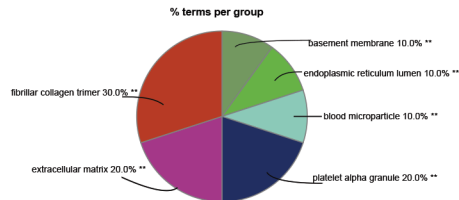

MF

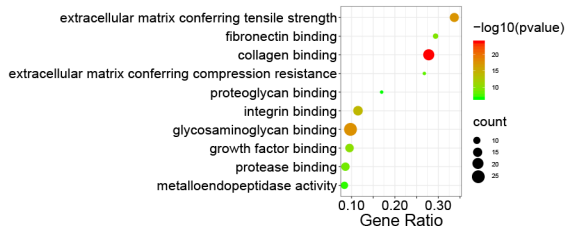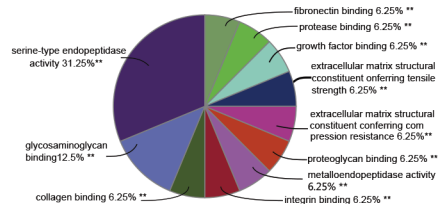

Supplement: Supplementary Figure 3 — Significant pathways influenced by PMEPA1 in TCGA.pdf. (A, B) Go and analysis of top 200 up-regulated genes. (A) The top 10 significant GO terms were listed for biological processes, cellular components, molecular functions. (B) Pie graph of specific Cluster. [file Image_3.pdf]

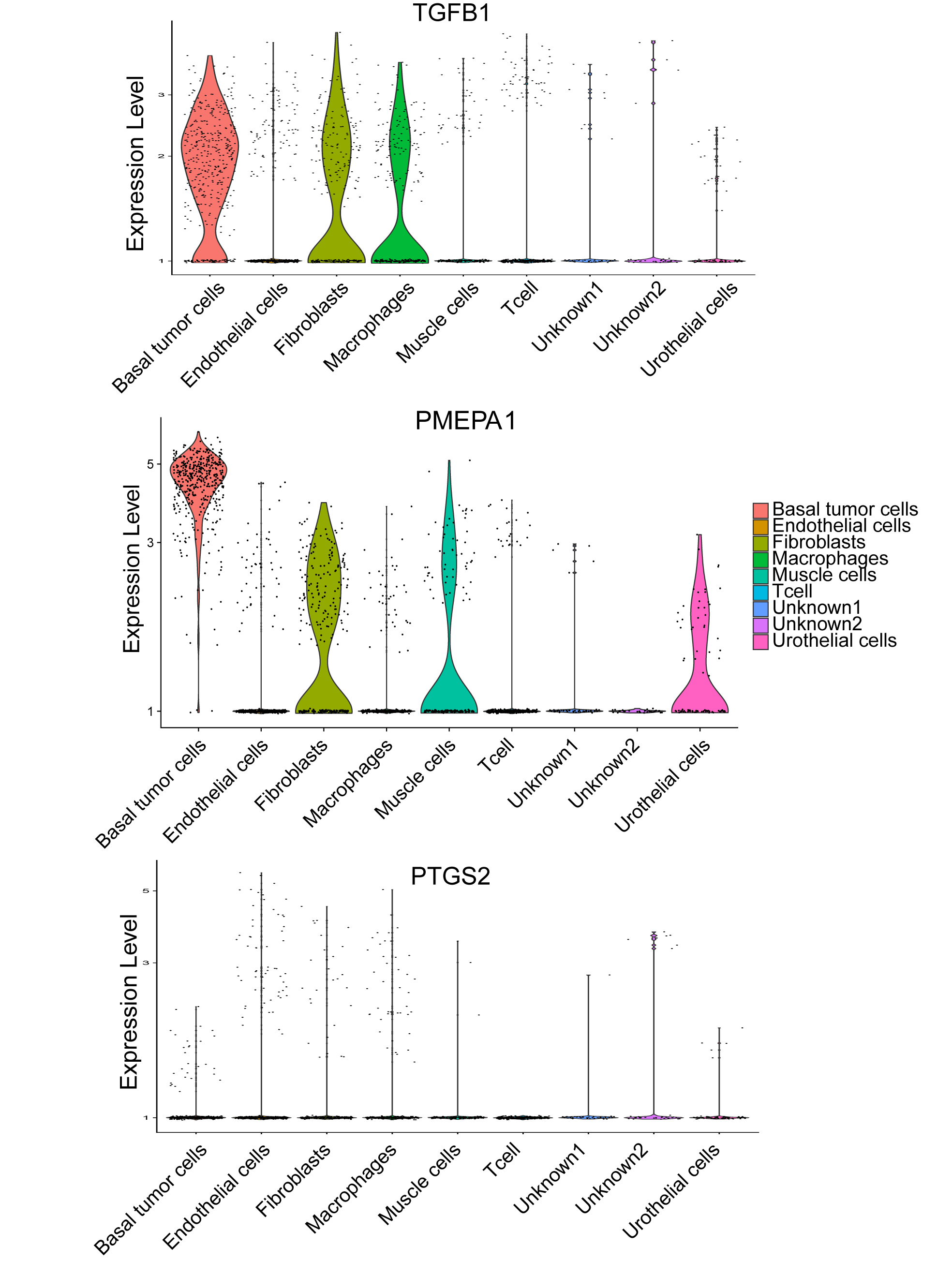

Supplement: Supplementary Figure 5 — The expression of TGFB1, PTGS2 and PMEPA1 in tumor cells, TAMs, CAFs, endothelial cells, T-cells, muscle cells, urothelial cells. The horizontal axis represents different cell types, and the vertical axis represents the expression level of PMEPA1, which were plotted via Seurat package of R software. All single-cell mRNA sequencing data was acquired from GSE145137. [file Image_5.tif]

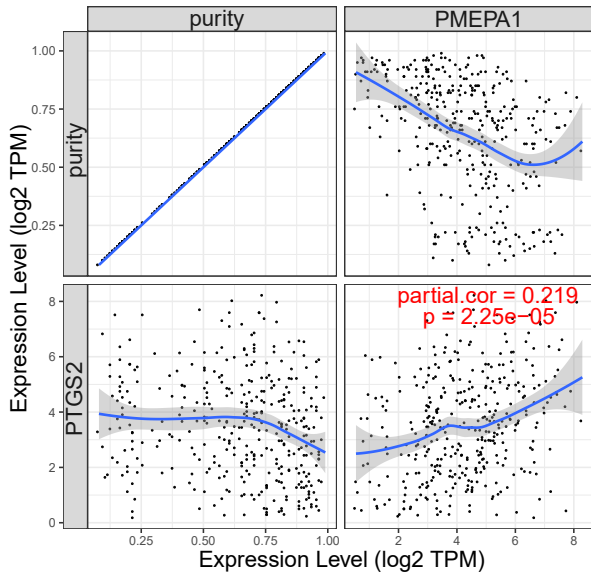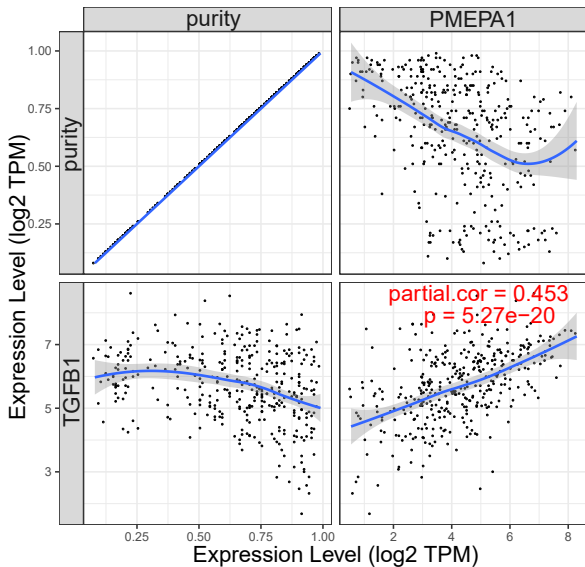

Supplement: Supplementary Figure 6 — Correlation analysis between TGFB1, PTGS2, and PMEPA1 in TIMER. The horizontal axis represents the expression level of PMEPA1 and the vertical axis represents the expression level of TGFB1 and PTGS2, The Partial.Cor and P-value were calculated after purity. Partial.Cor practical correlation coefficient. [file Image_6.pdf]

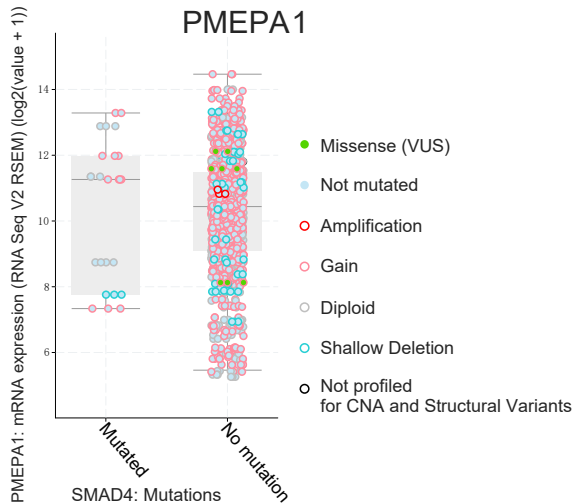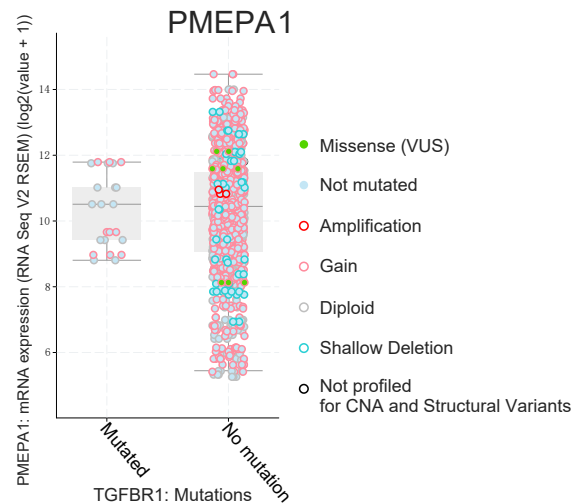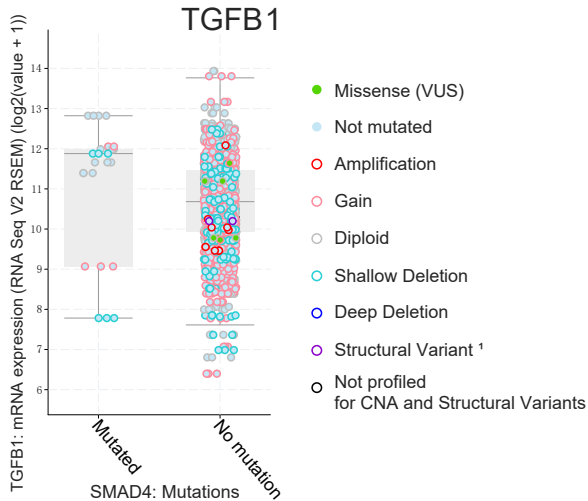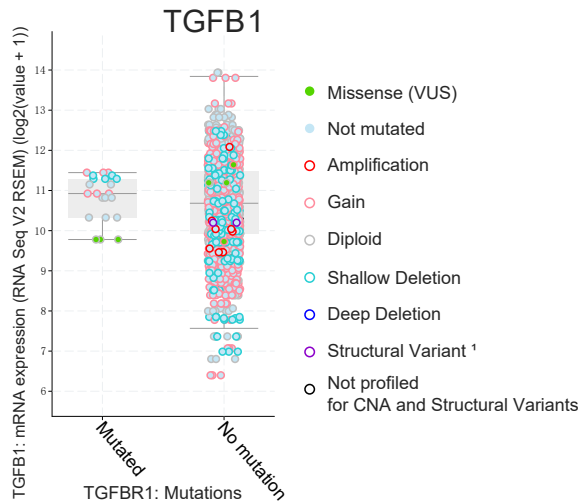

Supplement: Supplementary Figure 8 — The expression of PMEPA1 and TGFB1 in mutated/no mutation groups of SMAD4 and TGFBR. The horizontal axis represents the mutation states of SMAD4 and TGFBR and the vertical axis represents the expression levels of PMEPA1 and TGFB1, All the data was acquired from cBIoportal website (http://www.cbioportal.org). [file Image_8.pdf]
