# Supplementary material for: PMEPA1 Is a Prognostic Biomarker That Correlates With Cell Malignancy and the Tumor Microenvironment in Bladder Cancer
Source: Front Immunol. 2021 Oct 28;12:705086. doi: 10.3389/fimmu.2021.705086 (PMC8582246; doi:10.3389/fimmu.2021.705086)
Supplement: Supplementary Figure 4 — Correlation between PMEPA1 and the infiltration levels of 11 types of TIICs (TAMs, cancer associated fibroblasts (CAFs), myeloid-derived suppressor cells (MDSCs), monocytes, neutrophils, tregs, myeloid dendritic cells (DCs), NK cells, B cells, CD4+ T cells, CD8+ T cells) in pan-cancers using the TIMER database.pdf. The horizontal axis represents different tumor tissues, the vertical axis represents different TIICs using 8 independent algorithms, different colors represent correlation coefficients, and negative values represent negative correlations. A positive value represents a positive correlation. The stronger the correlation, the darker the color. P value of < 0.05 is considered statistically significant. [file Image_4.pdf]

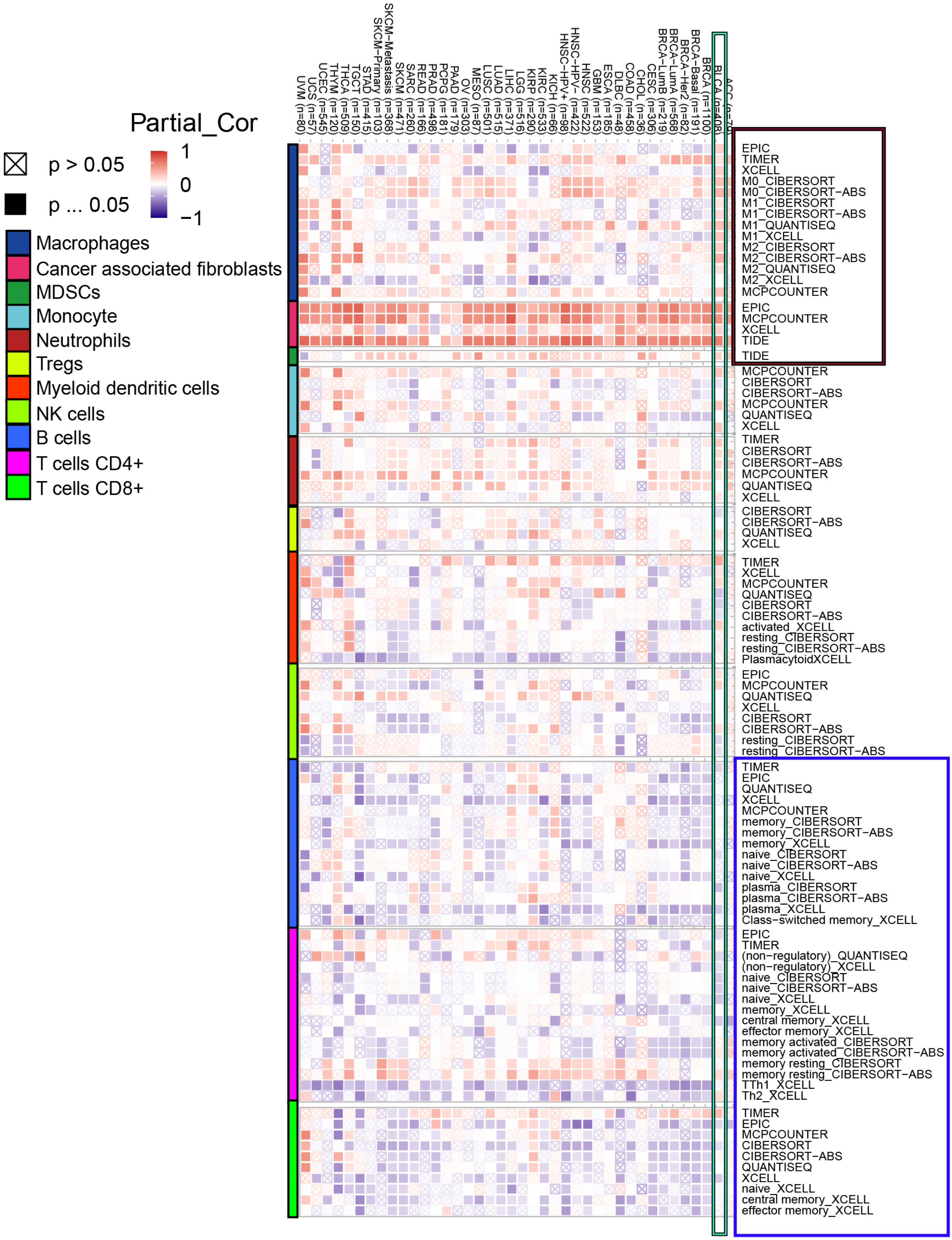

Partial_Cor
p > 0.05
p ... 0.05
1
0
−1
Macrophages
Cancer associated fibroblasts
MDSCs
Monocyte
Neutrophils
Tregs
Myeloid dendritic cells
NK cells
B cells
T cells CD4+
T cells CD8+
UVM (n=80)
UCS (n=57)
UCEC (n=545)
THYM (n=120)
THCA (n=509)
TGCT (n=150)
STAD (n=415)
SKCM−Primary (n=103)
SKCM−Metastasis (n=368)
SKCM (n=471)
SARC (n=260)
READ (n=166)
PRAD (n=498)
PCPG (n=181)
PAAD (n=179)
OV (n=303)
MESO (n=87)
LUSC (n=501)
LUAD (n=515)
LIHC (n=371)
LGG (n=516)
KIRP (n=290)
KIRC (n=533)
KICH (n=66)
HNSC−HPV+ (n=98)
HNSC−HPV− (n=422)
HNSC (n=522)
GBM (n=153)
ESCA (n=185)
DLBC (n=48)
COAD (n=458)
CHOL (n=36)
CESC (n=306)
BRCA−LumB (n=219)
BRCA−LumA (n=568)
BRCA−Her2 (n=82)
BRCA−Basal (n=191)
BRCA (n=1100)
BLCA (n=408)
ACC (n=7
EPIC
TIMER
XCELL
M0_CIBERSORT
M0_CIBERSORT−ABS
M1_CIBERSORT
M1_CIBERSORT−ABS
M1_QUANTISEQ
M1_XCELL
M2_CIBERSORT
M2_CIBERSORT−ABS
M2_QUANTISEQ
M2_XCELL
MCPCOUNTER
EPIC
MCPCOUNTER
XCELL
TIDE
TIDE
MCPCOUNTER
CIBERSORT
CIBERSORT−ABS
MCPCOUNTER
QUANTISEQ
XCELL
TIMER
CIBERSORT
CIBERSORT−ABS
MCPCOUNTER
QUANTISEQ
XCELL
CIBERSORT
CIBERSORT−ABS
QUANTISEQ
XCELL
TIMER
XCELL
MCPCOUNTER
QUANTISEQ
CIBERSORT
CIBERSORT−ABS
activated_XCELL
resting_CIBERSORT
resting_CIBERSORT−ABS
PlasmacytoidXCELL
EPIC
MCPCOUNTER
QUANTISEQ
XCELL
CIBERSORT
CIBERSORT−ABS
resting_CIBERSORT
resting_CIBERSORT−ABS
TIMER
EPIC
QUANTISEQ
XCELL
MCPCOUNTER
memory_CIBERSORT
memory_CIBERSORT−ABS
memory_XCELL
naive_CIBERSORT
naive_CIBERSORT−ABS
naive_XCELL
plasma_CIBERSORT
plasma_CIBERSORT−ABS
plasma_XCELL
Class−switched memory_XCELL
EPIC
TIMER
(non−regulatory)_QUANTISEQ
(non−regulatory)_XCELL
naive_CIBERSORT
naive_CIBERSORT−ABS
naive_XCELL
memory_XCELL
central memory_XCELL
effector memory_XCELL
memory activated_CIBERSORT
memory activated_CIBERSORT−ABS
memory resting_CIBERSORT
memory resting_CIBERSORT−ABS
TTh1_XCELL
Th2_XCELL
TIMER
EPIC
MCPCOUNTER
CIBERSORT
CIBERSORT−ABS
QUANTISEQ
XCELL
naive_XCELL
central memory_XCELL
effector memory_XCELL
