# Supplementary material for: PMEPA1 Is a Prognostic Biomarker That Correlates With Cell Malignancy and the Tumor Microenvironment in Bladder Cancer
Source: Front Immunol. 2021 Oct 28;12:705086. doi: 10.3389/fimmu.2021.705086 (PMC8582246; doi:10.3389/fimmu.2021.705086)
Supplement: Supplementary Figure 7 — The correlation and expression of PMEPA1, TGFB1, chemokines, and immune checkpoints in tumor cells (n=36), CAFs (n=273) and TAMs (n=283). (A) The correlation between PMEPA1 and TGFB1, chemokines and immune checkpoints in tumor cells, CAFs and TAMs. The horizontal axis represents the correlation coefficient and the vertical axis represents the different genes, the P-value is listed on the right. P value of < 0.05 is considered statistically significant. (B) The expression of PMEPA1, TGFB1, chemokines, and immune checkpoints in tumor cells, CAFs and TAMs. The horizontal axis represents different genes in tumor cells, CAFs and TAMs and the vertical axis represents the expression level, All single-cell mRNA sequencing data was acquired from GSE145137. [file Image_7.pdf]

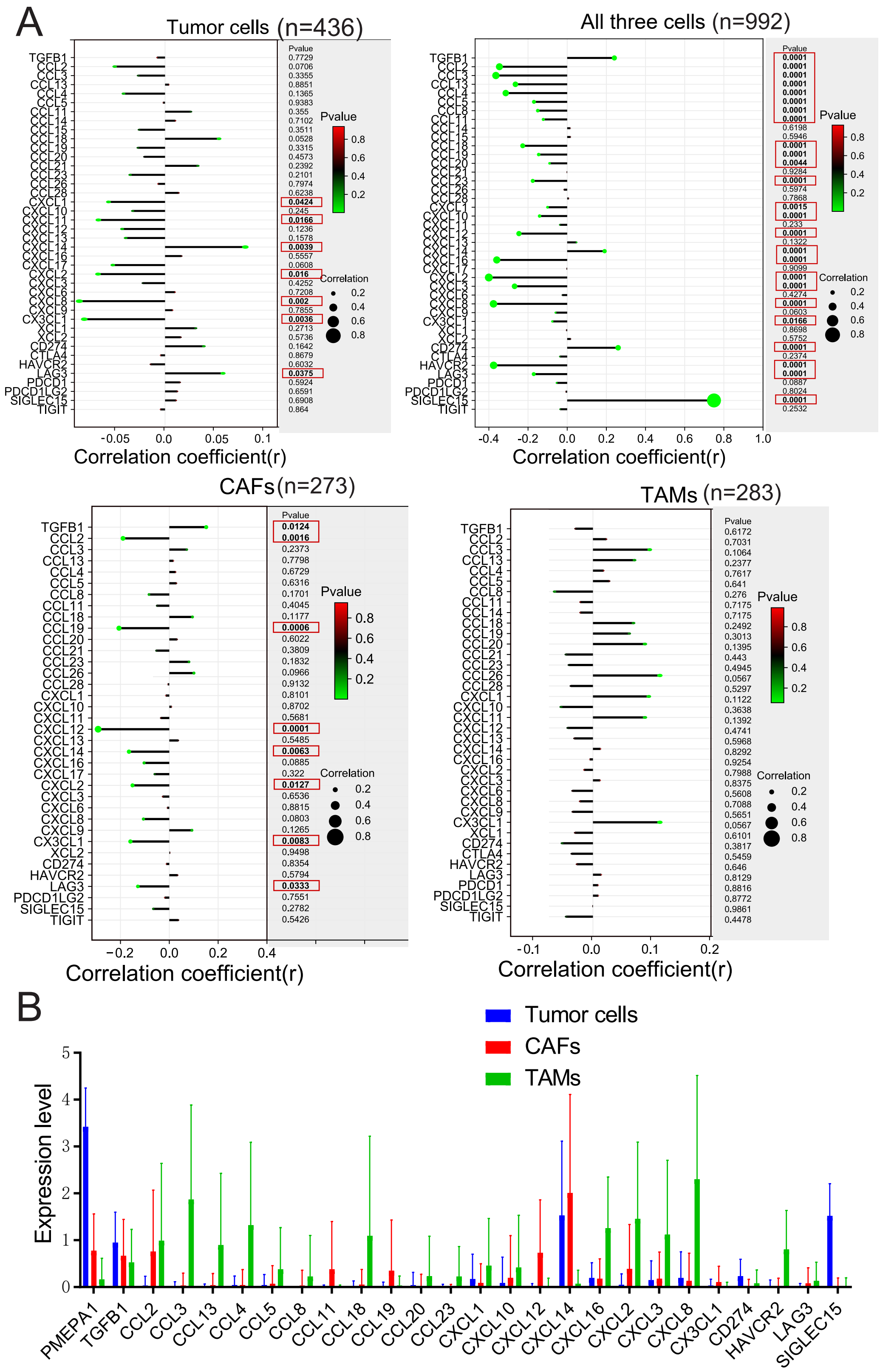

A
Tumor cells (n=436)
TGFB1
CCL2
CCL3
CCL13
CCL4
CCL5
CCL11
CCL14
CCL15
CCL18
CCL19
CCL20
CCL21
CCL23
CCL26
CCL28
CXCL1
CXCL10
CXCL11
CXCL12
CXCL13
CXCL14
CXCL16
CXCL17
CXCL2
CXCL3
CXCL6
CXCL8
CXCL9
CX3CL1
XCL1
XCL2
CD274
CTLA4
HAVCR2
LAG3
PDCD1
PDCD1LG2
SIGLEC15
TIGIT
Pvalue
0.7729
0.0706
0.3355
0.8851
0.1365
0.9383
0.355
0.7102
0.3511
0.0528
0.3315
0.4573
0.2392
0.2101
0.7974
0.6238
0.0424
0.245
0.0166
0.1236
0.1578
0.0039
0.5557
0.0608
0.016
0.4252
0.7208
0.002
0.7855
0.0036
0.2713
0.5736
0.1642
0.8679
0.6032
0.0375
0.5924
0.6591
0.6908
0.864
Pvalue
0.8
0.6
0.4
0.2
Correlation
0.2
0.4
0.6
0.8
-0.05
0.0
0.05
0.1
Correlation coefficient(r)
All three cells (n=992)
TGFB1
CCL2
CCL3
CCL13
CCL4
CCL5
CCL8
CCL11
CCL14
CCL15
CCL18
CCL19
CCL20
CCL21
CCL23
CCL26
CCL28
CXCL1
CXCL10
CXCL11
CXCL12
CXCL13
CXCL14
CXCL16
CXCL17
CXCL2
CXCL3
CXCL6
CXCL8
CXCL9
CX3CL1
XCL1
XCL2
CD274
CTLA4
HAVCR2
LAG3
PDCD1
PDCD1LG2
SIGLEC15
TIGIT
Pvalue
0.0001
0.0001
0.0001
0.0001
0.0001
0.0001
0.0001
0.0001
0.6198
0.5946
0.0001
0.0001
0.0044
0.9284
0.0001
0.5974
0.7868
0.0015
0.0001
0.233
0.0001
0.1322
0.0001
0.0001
0.9099
0.0001
0.0001
0.4274
0.0001
0.0603
0.0166
0.8698
0.5752
0.0001
0.2374
0.0001
0.0001
0.0887
0.8024
0.0001
0.2532
Pvalue
0.8
0.6
0.4
0.2
Correlation
0.2
0.4
0.6
0.8
-0.4
-0.2
0.0
0.2
0.4
0.6
0.8
1.0
Correlation coefficient(r)
CAFs (n=273)
TGFB1
CCL2
CCL3
CCL13
CCL4
CCL5
CCL8
CCL11
CCL18
CCL19
CCL20
CCL21
CCL23
CCL26
CCL28
CXCL1
CXCL10
CXCL11
CXCL12
CXCL13
CXCL14
CXCL16
CXCL17
CXCL2
CXCL3
CXCL6
CXCL8
CXCL9
CX3CL1
XCL2
CD274
HAVCR2
LAG3
PDCD1LG2
SIGLEC15
TIGIT
Pvalue
0.0124
0.0016
0.2373
0.7798
0.6729
0.6316
0.1701
0.4045
0.1177
0.0006
0.6022
0.3809
0.1832
0.0966
0.9132
0.8101
0.8702
0.5681
0.0001
0.5485
0.0063
0.0885
0.322
0.0127
0.6536
0.8815
0.0803
0.1265
0.0083
0.9498
0.8354
0.5794
0.0333
0.7551
0.2782
0.5426
Pvalue
0.8
0.6
0.4
0.2
Correlation
0.2
0.4
0.6
0.8
−0.2
0.0
0.2
0.4
Correlation coefficient(r)
TAMs (n=283)
TGFB1
CCL2
CCL3
CCL13
CCL4
CCL5
CCL8
CCL11
CCL14
CCL18
CCL19
CCL20
CCL21
CCL23
CCL26
CCL28
CXCL1
CXCL10
CXCL11
CXCL12
CXCL13
CXCL14
CXCL16
CXCL2
CXCL3
CXCL6
CXCL8
CXCL9
CX3CL1
XCL1
CD274
CTLA4
HAVCR2
LAG3
PDCD1
PDCD1LG2
SIGLEC15
TIGIT
Pvalue
0.6172
0.7031
0.1064
0.2377
0.7617
0.641
0.276
0.7175
0.7175
0.2492
0.3013
0.1395
0.443
0.4945
0.0567
0.5297
0.1122
0.3638
0.1392
0.4741
0.5968
0.8292
0.9254
0.7988
0.8375
0.5608
0.7088
0.5651
0.0567
0.6101
0.3817
0.5459
0.646
0.8129
0.8816
0.8772
0.9861
0.4478
Pvalue
0.8
0.6
0.4
0.2
Correlation
0.2
0.4
0.6
0.8
-0.1
0.0
0.1
0.2
Correlation coefficient(r)
B
Tumor cells
CAFs
TAMs
Expression level
0
1
2
3
4
5
PMEPA1
TGFB1
CCL2
CCL3
CCL13
CCL4
CCL5
CCL8
CCL11
CCL18
CCL19
CCL20
CCL23
CXCL1
CXCL10
CXCL12
CXCL14
CXCL16
CXCL2
CXCL3
CXCL8
CX3CL1
CD274
HAVCR2
LAG3
SIGLEC15
